# A Humanized Clinically Calibrated Quantitative Systems Pharmacology Model for Hypokinetic Motor Symptoms in Parkinson’s Disease

**DOI:** 10.3389/fphar.2016.00006

**Published:** 2016-02-02

**Authors:** Patrick Roberts, Athan Spiros, Hugo Geerts

**Affiliations:** ^1^In Silico BiosciencesBerwyn, PA, USA; ^2^Washington State UniversityVancouver, WA, USA; ^3^Perelman School of Medicine, University of PennsylvaniaPhiladelphia, PA, USA

**Keywords:** Parkinson’s disease, systems pharmacology, drug treatment

## Abstract

The current treatment of Parkinson’s disease with dopamine-centric approaches such as L-DOPA and dopamine agonists, although very successful, is in need of alternative treatment strategies, both in terms of disease modification and symptom management. Various non-dopaminergic treatment approaches did not result in a clear clinical benefit, despite showing a clear effect in preclinical animal models. In addition, polypharmacy is common, sometimes leading to unintended effects on non-motor cognitive and psychiatric symptoms. To explore novel targets for symptomatic treatment and possible synergistic pharmacodynamic effects between different drugs, we developed a computer-based Quantitative Systems Pharmacology (QSP) platform of the closed cortico-striatal-thalamic-cortical basal ganglia loop of the dorsal motor circuit. This mechanism-based simulation platform is based on the known neuro-anatomy and neurophysiology of the basal ganglia and explicitly incorporates domain expertise in a formalized way. The calculated beta/gamma power ratio of the local field potential in the subthalamic nucleus correlates well (*R*^2^ = 0.71) with clinically observed extra-pyramidal symptoms triggered by antipsychotics during schizophrenia treatment (43 drug-dose combinations). When incorporating Parkinsonian (PD) pathology and reported compensatory changes, the computer model suggests a major increase in b/g ratio (corresponding to bradykinesia and rigidity) from a dopamine depletion of 70% onward. The correlation between the outcome of the QSP model and the reported changes in UPDRS III Motor Part for 22 placebo-normalized drug-dose combinations is *R*^2^ = 0.84. The model also correctly recapitulates the lack of clinical benefit for perampanel, MK-0567 and flupirtine and offers a hypothesis for the translational disconnect. Finally, using human PET imaging studies with placebo response, the computer model predicts well the placebo response for chronic treatment, but not for acute treatment in PD.

## Introduction

Drug development in CNS diseases is faced with a high rate of failure in clinical trials and PD is no exception.

While animal models are very good at elucidating the underlying molecular biology of individual targets and their interaction with a number of pathways, they are less predictive for clinical outcome of specific novel therapeutic interventions.

Important translational issues that are beyond the reach of animal models and affect the outcome of clinical trials include lack of attention to comedications, differences in affinity between identical targets in different species, the formation of unique human metabolites, human genotypes that interfere directly or indirectly with the primary pharmacology, and the appropriate patient population (for a review see, Geerts, 2009). Interestingly, while the majority of clinical trial failures are due to lack of efficacy, often unexpected motor-related side-effects are observed in clinical trials for new antipsychotics such as JNJ37822681, a highly selective low-affinity dopamine D_2_ antagonist (Geerts et al., 2012) or the PDE-10A inhibitor (Zhang, 2010).

A number of novel *in silico* technologies as support for drug discovery and development have been proposed (Butte and Ito, 2012). Almost all of them are based upon statistical data-mining and pattern recognition of large databases of drugs and their clinical effects and rely upon correlation rather than causation.

Quantitative system pharmacology is a logical combination of quantitative PharmacoDynamic modeling (Geerts et al., 2013b) with the rich information derived from Systems Biology (Sorger and Schoeberl, 2012). In the case of CNS diseases we can build on the extensive expertise of computational neurosciences that has been around for over 60 years since the seminal work of Hodgkin and Huxley (Hodgkin and Huxley, 1952). In a recent Big Science initiative, the Blue Brain project (Markram, 2012) simulated the time-dependent membrane potential changes and action potentials in a detailed computer model of a human microcolumn, containing over 200 different cell types and a wide variety of voltage-gated ion channels. However, although such a model can provide increased understanding of the human neurobiology, it lacks aspects that could make it useful for CNS drug discovery, such as application of human pathology parameters from imaging and post-mortem studies; neuropharmacological targets of CNS-active drugs, a proper description of target engagement of these drugs and a calibration using retrospective and historical clinical data.

We therefore developed, calibrated and validated a version of a computational neurosciences model that includes these quantitative pharmacological aspects in the framework of neuronal circuits to make it more actionable for pharmaceutical Research and Development. Such an approach falls under the definition of QSP (van der Graaf and Benson, 2011; Sorger and Schoeberl, 2012). The current platform covers clinical readouts in schizophrenia (Geerts et al., 2012, 2013a, 2015; Spiros et al., 2012; Liu et al., 2014), Parkinson’s (Spiros et al., 2013), and Alzheimer’s disease (Roberts et al., 2012; Nicholas et al., 2013).

This report presents a computer-based disease-modeling platform of the basal ganglia for assessing the motor-symptoms observed in PD that is fully constrained by clinical data on rigidity and dyskinesia.

While the original hypothesis of Parkinson’s pathology was focused on activation imbalance in direct vs. indirect pathways (Miller and DeLong, 1988), recent studies using information obtained from electrodes implanted in basal ganglia regions point to the encoding of information in local field potential oscillatory behavior in specific basal ganglia regions, notably the STN (Alonso-Frech et al., 2006; Little et al., 2013). We therefore define the readout of our computer model as the ratio of the power contained in different spectral bands of the STN, notably in the beta over gamma band.

We start with calibrating the side-effects liability of antipsychotics in schizophrenia which covers a broad range of receptor couplings in the model beyond the dopaminergic system, because these drugs affect many different receptors. Obviously in this case, we implement the appropriate hyperdopaminergic state in the basal ganglia, derived from imaging studies in human patients (Abi-Dargham et al., 2000). By optimizing the correlation between the outcome of historical trials and the model outcomes, the biological coupling parameters in the computer model can be calibrated. With this list of optimal coupling parameters, we can then proceed to implementing Parkinsonian pathology (hypodopaminergic state) and testing the effect of therapeutic interventions. Using this two-pronged approach ensures we capture a wide dynamic range of the biological processes that drive motor behavior.

In this way, such a QSP platform can become very useful for CNS R&D in schizophrenia motor side-effects, Parkinson’s and Huntington’s diseases. By using such a humanized computer model, we aim to increase the predictability of clinical outcomes by running ‘virtual’ human patient trials allowing one to determine the impact of comedications and genotypes on the dose-response of a novel investigative drug. The input for such a ‘virtual’ patient trial will be the complete pharmacology against human targets of the drug under investigation and the nature and dose of other comedications. The output of the computer model is a dose-dependent *in silico* biomarker (here the ratio of the power contained in beta and gamma spectral bands of the STN local field potential) that is calibrated against the clinical UPDRS scale. We can then estimate an anticipated clinical effect size and optimize clinical trial design for a new therapeutic intervention or estimate the impact of comedications on the dose-response of a new therapeutic intervention.

## Materials and Methods

### Receptor Competition Model

Proper target engagement is a key issue in clinical development. Often a therapeutic window is defined as the ratio of the dose for a side-effect vs. the dose of a clinical effect. Therefore, we simulate target engagement of the drug at a specific dose using quantitative PET imaging displacement studies with specific radiotracers, where available. In the case of antipsychotics, D_2_-specific tracers, such as raclopride, IBZM, or fallypride are often used. We developed a receptor competition synapse model that simulates the competition between neurotransmitter, parent and active metabolite and tracer for dopaminergic, serotonergic, adrenergic, or cholinergic post-synaptic receptors (Spiros et al., 2010). The synaptic model reflects the human dynamics of the various neurotransmitters by calibrating the presynaptic autoreceptor coupling physiology with fast cyclic voltammetry data from rodents and primates and constrains it subsequently by human imaging data. This allows one to derive free levels of neurotransmitter and presynaptic firing frequencies leading to basal receptor activation levels in healthy subjects and patients. Of importance for this paper, this leads to a substantial difference between rodent and human dopaminergic dynamics (Spiros et al., 2010).

### Neural Microcircuit Model

Parameters of the neural microcircuit component include channel kinetics, structure, conductances, and receptor effects. The *channel kinetic parameters* determine the time course and voltage sensitivity of membrane and synaptic currents and have been well-characterized by physiological experimentation *in vitro*. *Structural parameters* define the dimensions of compartments that represent neurons and are determined by anatomical data and the minimal number of compartments that are necessary to simulate the physiological activity of the microcircuit. These parameters also define the connectivity between neurons in the microcircuit and are constrained by anatomical data. In addition, neuronal cells project to their afferent regions based on neuro-anatomical connectivity data. The parameters for maximum *conductances* of membrane and synaptic currents are adjusted for the initial tuning of each neuronal circuit model to accurately simulate neural activity at the system’s level, often measured in primates by single-cell electrophysiology or local field potentials from deep-brain recording in patients or data from human EEG and fMRI. These parameters are constrained by electrophysiological studies of membrane current densities and synaptic currents, but are also dependent on the specific implementation of the compartmental models to represent biophysical microcircuits.

*The change in receptor activation* introduces pharmacology in the models and allows one to calibrate the models with human clinical data. The effects of neural modulators such as dopamine and serotonin are implemented by coupling the activation of receptors to changes in membrane and synaptic currents. These effects tend to modulate, rather than drive, the overall activity of the network. Reasonably small changes in receptor activation will not cause drastic change to the overall systems level behavior, but will produce measurable changes in the model readouts. Therefore, we consider the modulation of receptors by pharmacological agents to be a perturbation of the state of the system, whether that state is normal or pathological. We can thus use a first-order (linear) approximation of the changes caused by pharmacology to alter the effects of receptor activation.

### Neuronal Models Using the NEURON Simulation Package

Each cell type is modeled with membrane conductances to simulate their functional role in the circuit, and for effects of receptor activations caused by the pharmacology to change the spiking activity. Each compartment of each model neuron obeys the membrane current balance equation of the Hodgkin-Huxley formalism (Hodgkin and Huxley, 1952). The membrane potential, V, is computed by numerically integrating the equation for each compartment CdV/dt = ∑ g_a_(V-E_a_)+I_ex_, where C is the membrane capacitance, g_a_ is the ionic conductance of a specific type ion channel, and E_a_ is the reversal potential of that ion channel. The sum is over all types of ion conductances in each model compartment, and I_ex_ represents an externally applied current from synaptic currents.

### Specific Model of Subcortical Circuitry to Predict Effects of Drug Actions on Clinical Scales

**FIGURE 1:**
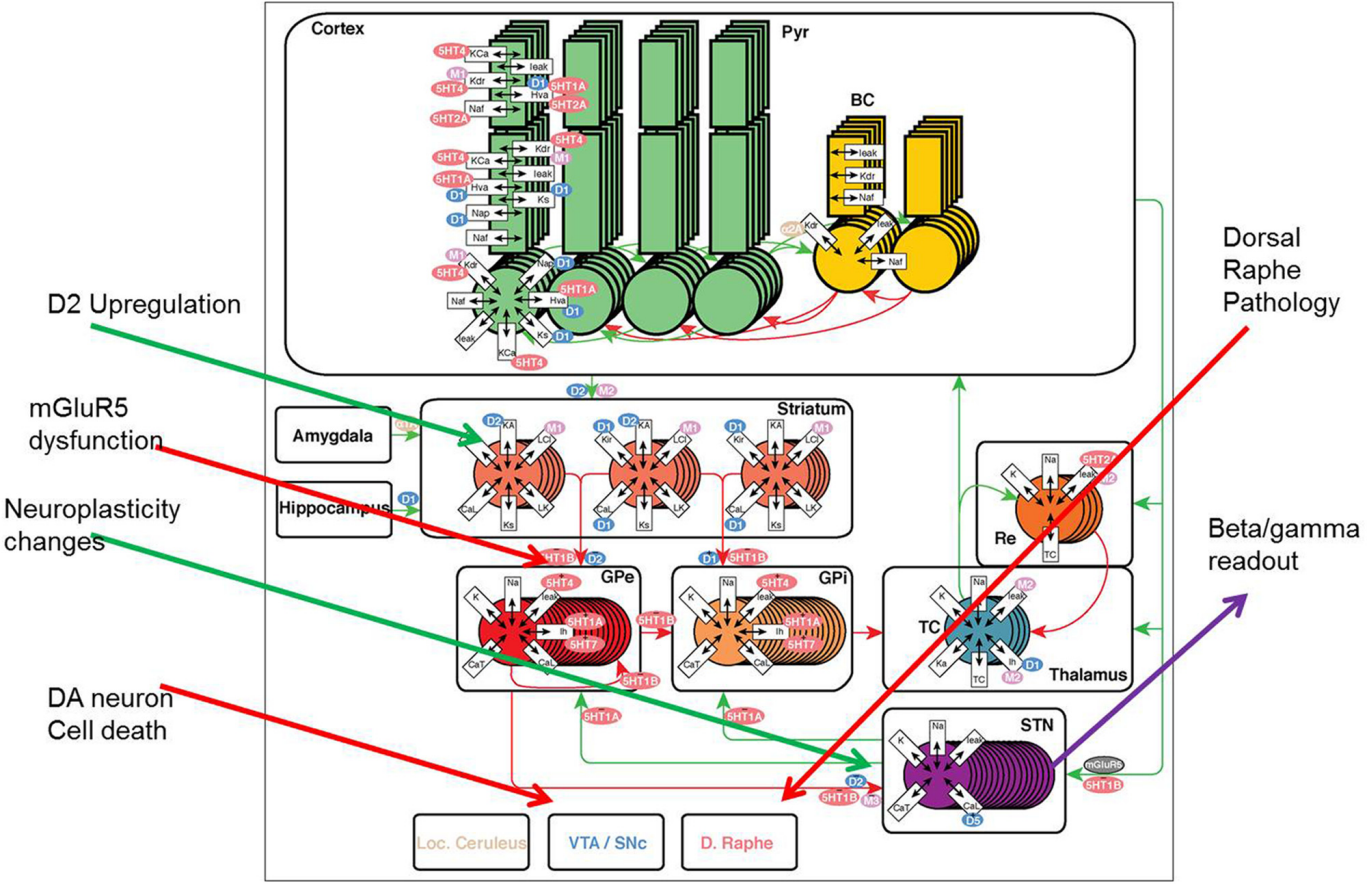
**The model framework for basal ganglion, cortex, and thalamus.** Two types of medium spiny neurons in the striatum, D_1_ and D_2_, project inhibitory (red) synapses to GP_i_ (direct pathway) and GP_e_ (indirect pathway) respectively. The GPe neurons are reciprocally coupled to themselves and the subthalamic nucleus (STN). The STN projects excitatory (green) synapses to the GP_i_ and the GP_i_ projects inhibitory connections to the thalamus. Thalamocortical (TC) neurons excite reticular (Re) neurons that reciprocally inhibit TC. Sensory input excites TC and TC projects to the cortex containing pyramidal cells (Pyr) and inhibitory basket cells (BC). All cell types receive background excitatory and inhibitory fluctuating inputs that represent random synaptic activity. White rectangles represent membrane currents and colored ovals represent receptor types coupled to the membrane and synaptic currents. See text for currents and receptor types. PD pathology (red arrows) is implemented using DA neuronal cell death, mGluR_2-3_ dysfunction at the MSN-GP_e_ (indirect pathway) connection and dorsal raphe changes; while compensatory changes (green arrows) include changes in the GP_e_-STN coupling and DA synapse modification, such as D_2_R upregulation and DAT downregulation.

The model of a closed cortico-striatal-thalamo-cortical circuitry consists of three components: striatum, STN-GP circuitry, and thalamo-cortical circuitry (**Figure 1**) and is based on a previous model (Pirini et al., 2009) where we have added all relevant neuromodulator receptor effects and subsequently implemented PD pathology (see below). The STN-GP circuitry consists of two segments of the GP_e_ and GP_i_ and the STN. The thalamus model is extended from (Bazhenov et al., 1998) where specific receptor effects of interest for pharmaceutical research were added as part of a thalamo-cortical model.

#### Striatum Component

The striatum model simulates the processing capacity of MSNs in the ventral striatum or nucleus accumbens (Spiros et al., 2012). The model calculates the excitability of the MSN, the major GABA-ergic cell type in the nucleus accumbens, when driven by afferent cortical projections. We simulate two types of MSNs: D_1_ cells that project to the direct pathway and D_2_ cells that project to the indirect pathway.

In the D_1_+ cells of the direct pathway, D_1_ receptors mostly affect the K_ir_ channel (Kuzhikandathil and Oxford, 2002), while in D_2_+ cells (indirect pathway), D_2_ receptor activation mainly affects the A-type K+ current (Gabel and Nisenbaum, 1998; Falk et al., 2006). In both MSN, D*_2_* receptor activity modulates the presynaptic Glu release on the afferent cortical fibers (Bamford et al., 2004).

#### STN-GP Circuitry Component

The model for the direct and indirect pathways is based on the (Rubin and Terman, 2004) model of STN, GP_i_, and GP_e_, and its extension (Pirini et al., 2009). Each nucleus contains 16 neurons with the following membrane currents: sodium current (Na^+^), delayed rectifier potassium current (K_dr_^+^), T-type calcium current (Ca_T_^++^), L-type calcium current (Ca_L_^++^) and a leak current. Receptor modulation of these neurons by receptors of the dopamine, serotonin, acetylcholine, and adrenergic systems will be obtained from the literature.

The STN, GP_e_, and GP_i_ population is divided in two groups of eight cells each. The D_2_-type MSN cells from the striatum synaptically connects to the GP_e_ neurons with a GABA current and the D_1_-type MSN cells has a GABA synapse onto the GP_i_ neurons. Each GP_e_ cell receives inhibitory input from three other GP_e_ cells in addition to excitatory input from three randomly chosen STN cells. Each STN cell receives inhibitory input from three GP_e_ cells. Each GP_i_ cell receives inhibitory input from one GP_e_ neuron and excitatory input from one STN neuron. All the TC cells of the thalamus receive inhibitory input from eight GP_i_ cells. Synaptic couplings are implemented as shown in **Figure 1**.

#### Thalamo-Cortical Component

The thalamus model is based on the circuitry and cellular properties (see below) with four thalamocortical neurons (TC) that are excitatory, glutamatergic relay neurons that pass sensory information to the cortex model described in (Bazhenov et al., 1998). The four reticular neurons (Re) are inhibitory, GABAergic feedback neurons that receive inputs from, and inhibit, TC neurons. The synaptic interaction between these neuronal types, and their intrinsic membrane properties, leads to oscillations and suppression of multiple input signals.

The spiking property of TC neurons is caused by a fast sodium channel, Na^+^ (for review, see Traub et al., 1991), a fast potassium channel, K^+^ (Traub et al., 1991), a low-threshold Ca^++^ channel, iTC (Huguenard and McCormick, 1992), a hyperpolarization-activated cation channel, Ih (McCormick and Huguenard, 1992; Destexhe et al., 1998), a potassium A channel, Ka^+^ (Huguenard et al., 1991), and a potassium leak channel (McCormick and Huguenard, 1992). For an Re cell, we included a fast sodium channel, Na^+^, a fast potassium channel, K^+^, a low-threshold Ca^++^ channel, iTC, and a potassium leak channel. All parameters (channel kinetics, current densities, morphology, and synaptic strengths) were set at the values given in Bazhenov et al. (1998). Muscarinic M_2_ receptor activation increases the maximum conductance of h channels in thalamocortical cells and the maximum conductance of leak channels in reticular cells, similar to the effect of serotonergic 5-HT2A receptor activation.

The cortex contains pyramidal cells (Pyr) and inhibitory basket cells (BCs) and is derived from the model described for working memory (Roberts et al., 2012; Geerts et al., 2013a). Basically this version consists of 40 two-compartment pyramidal cells and 20 two-compartment GABA inhibitory neurons. 60% of the interneurons synapse on pyramidal cells, the remaining part forming a microcircuit. The pyramidal cells are driven by the thalamic projections and they project to the MSN neurons in the striatum for both direct and indirect pathway and to the STN for the hyperdirect pathway.

#### Synaptic Currents

The synaptic connections are based on the kinetics of AMPA, NMDA, GABA-A, and mGluR currents. Excitatory synapses include both AMPA (Destexhe, 1994; Jahn et al., 1998; Myme et al., 2003) and NMDA (Jahr and Stevens, 1990; Destexhe, 1994; Dalby and Mody, 2003) currents. Parameters include maximal inward depolarizing conductance *(g)*, rise time constant (*t*_rise_), and decay time constant (*t*_decay_). Reversal potentials for these excitatory conductances have been set to 0 mV (Traub et al., 2004). The following equation describes the Na^+^ conductance (*g*_glu_) of both AMPA and NMDA receptors used in this model [Mg^++^block for NMDAR not shown (Jahr and Stevens, 1990)]: *g*_glu_*(t)* = < *g* > (exp(-*t*/*t*_decay_) – exp(-*t*/*t*_rise_). We represent the effects of mGluR stimulation with a slow, depolarizing current (Anwyl, 1999; Sidiropoulou et al., 2009). Inhibitory chemical synapses represent GABA-A receptor currents (Destexhe, 1994; Galarreta and Hestrin, 1997; Nusser et al., 1998; Destexhe et al., 2001) using a similar scheme as excitatory synapses, with the GABA-A (chloride) reversal potential and kinetics associated with each cell type. Each model neuron receives fluctuating currents to simulate background synaptic bombardment by excitatory and inhibitory neurons (Destexhe et al., 2001).

#### Power Spectrum Analysis

Synaptic currents are used to calculate frequency band activity of local field potentials. To extract the EEG signal from the simulated network activity, we assume that synaptic currents in pyramidal cells are the major contributing dipole that generates field potentials (Nunez and Srinivasan, 2006), because their large number dominates the synaptic currents in other types of cells. Evoked potentials are simulated by excitatory synaptic inputs from a burst of spikes transmitted via AMPA and NMDA currents to represent sensory inputs (Moxon et al., 2003a,b; Zachariou et al., 2008). Gamma power (γ) is then calculated as the integral of the gamma band (60–100 Hz), while beta-power (β) is calculated as the integral over the explanded spectrum (12–50 Hz) for less variability in a 2.5 s simulation than a more properly defined spectrum of 10–40 Hz.

### Receptor Effects, Pharmacology, and Disease State

#### Receptor Effects

The receptor effects are coupled to membrane conductances and synaptic currents. The receptor competition model (Spiros et al., 2010, 2012) calculates the occupancy and activation percentage of each receptor using the appropriate affinities of the different drugs at their functional intrasynaptic concentration, which in the case of antipsychotics, will be derived from human imaging studies with the appropriate PET radiotracers. The percent change in the maximum conductance will determine how the membrane and synaptic currents change as a result of each drug-dose combination. The modulation of voltage-gated ion channels will lead to a change in spiking activity of the model and alter the properties of oscillatory readouts.

#### Implementation of Receptor Pharmacology

Serotonin receptors modulate currents depending on location and magnitude. Adrenergic receptor activation enhances K^+^ conductance at the soma of interneurons, decreasing GABA neuron excitability and regulates transmission at glutamatergic, but not at GABAergic synapses. Cholinergic effects in pyramidal cells are implemented by coupling the computed activation of M_1_ receptors to potassium and leak conductances in the model. Nicotinic receptors enhance GABA neurotransmission and glutamate release in pyramidal and interneuron NMDA and AMPA conductances. For a full list of the interactions with the appropriate references see Supplementary Table S1.

#### Implementation of the Schizophrenia Pathology

Schizophrenia pathology is introduced in the model as changes in the level of striatal dopamine, based upon human imaging studies (Abi-Dargham et al., 2000) and other receptor densities changes (Geerts et al., 2012; Spiros et al., 2012).

#### Implementation of Parkinson Pathology

We introduce PD pathology as a general decrease in dopamine release for both direct and indirect pathways (reflecting SN pathology of DA neurons). Reduced DA tone affects considerably the beta/gamma power ratio in the STN as measured from deep-brain recordings in PD patients (Tan et al., 2013). A compensatory upregulation of D_2_R and downregulation of DAT has been observed in to post-mortem and imaging studies (Piggott et al., 1999). Selective upregulation of D_2_R was implemented with a function that adjusted the fraction of high affinity dopamine D_2_R from 75% (at normal dopamine release) to 100% when DA release is limited to 5% of normal. Downregulation of DAT was implemented using changes in the half-life of synaptic DA from 50 ms (at normal dopamine release) to 70 ms when DA release nears its theoretical limit of 0. The selective loss of bidirectional plasticity at the MSN-GP_e_ and GP_e_-STN boundary (Thiele et al., 2014) was implemented by changing the coupling factor at the boundary of these regions. Finally changes in 5-HT tone on receptors in basal ganglia (Miguelez et al., 2014) affect the activation levels of all the different 5-HTR. **Figure 1** gives an overview of the Parkinsonian pathological changes and compensatory modifications.

### Calibration of QSP Model with Clinical Data

#### Motor Side Effects in Schizophrenia Patients during Treatment with Antipsychotics

Using PubMed searches we generated a clinical database of 76 tested drug-dose combinations derived from peer-reviewed publications of double-blind therapeutic interventions in schizophrenia that covers the period from 1988 to today (see Supplementary Table S2). The clinical data are for short-term (4–12 weeks) studies on stable schizophrenia patients that are switched to investigative antipsychotic treatment or placebo after a 2 weeks washout. The database contains 27 different antipsychotics and cover over 30,000 patients, although we only have access to group-average clinical readouts. None of the studies test augmentation therapy with antipsychotics but most of them (62%) compare a new drug with an existing active comparator, the remainder of the studies includes a placebo arm. We identified 327 clinical experimental conditions (including placebo); for each drug-dose combination, a weighted average is calculated using the actual number of patients in that study resulting in 76 drug-dose combinations; 43 of which are report on the fraction of patients needing anticholinergic medication for treatment of their Parkinsonian Extra-Pyramidal motor-side effects used in this calibration.

#### Clinical Calibration of Parkinson’s Disease

Clinical data with regard to their effect on the UPDRS scale are available for 24 different drugs including dopaminergic agents, NMDA antagonists, AMPAkines, Adenosine A_2A_ agonists, MAO-B inhibitors, 5-HT_2A_ antagonists, anticholinergics, SERT inhibitors and COMT inhibitors leading to 34 drug-dose combinations on the UPDRS Part III motor score with 12 corresponding placebo effects (see also Supplementary Table S3). Most patients are from Caucasian origin with a somewhat higher fraction of men. We ended up with 163 individual drug-dose combinations (including placebo) with 103 (63%) in combination with optimal dose of L-DOPA, 17 (10%) in combination with other Parkinson’s medication (but not L-DOPA), 35 (22%) stand-alone and 8 (5%) cross-over studies. 59 conditions (36%) were studied in early disease state (<5 years), 47 (29%) conditions in moderate disease (5–10 years) and 57 (35%) in severe disease (>10 years). Study duration ranged from acute (30 min) to long-term (113 weeks), but for the calibration we restricted the data-set to maximal duration of 12 weeks. Although the total number of patients in this database is over 17,000, we have only access to group-average values for clinical outcomes. Note that all conditions did not report on the same clinical scale. UPDRS Part III had 63 drug-dose combinations, the change in OFF time had 41 drug-dose combinations, followed by UPDRS II (39), change in UPDRS Total (29) and change in ON time (21). We will simulate these detailed therapeutic interventions in the model and compare the calculated model outcome (beta/gamma power of local field potentials in the STN) to actual changes in clinical scales.

## Results

### Calibration of the Network with Parkinsonian Extra-Pyramidal Side-Effect Profile of Antipsychotics

Antipsychotics do have a rich pharmacology and affect many receptors. Because the extra-pyramidal side-effect is similar to Parkinson’s symptoms of bradykinesia and rigidity, calibrating the computer model with these clinical data will optimize the seven coupling factors (see below) for a wide range of receptor couplings. For each of the 43 antipsychotic-dose combinations we first calculate the striatal intrasynaptic functional concentration of the antipsychotic parent molecule and its metabolite where available from the observed D_2_R specific PET imaging tracer displacement in schizophrenia patients (Spiros et al., 2012). This ensures we use the actual functional brain concentration for the active moiety. Using the appropriate affinities of the antipsychotic and its metabolites for all GPCR, we can then calculate the changes in receptor activation levels for all dopaminergic, serotonergic, adrenergic, and cholinergic receptors in a schizophrenia pathology environment that are affected by that specific drug-dose antipsychotic treatment.

**FIGURE 2:**
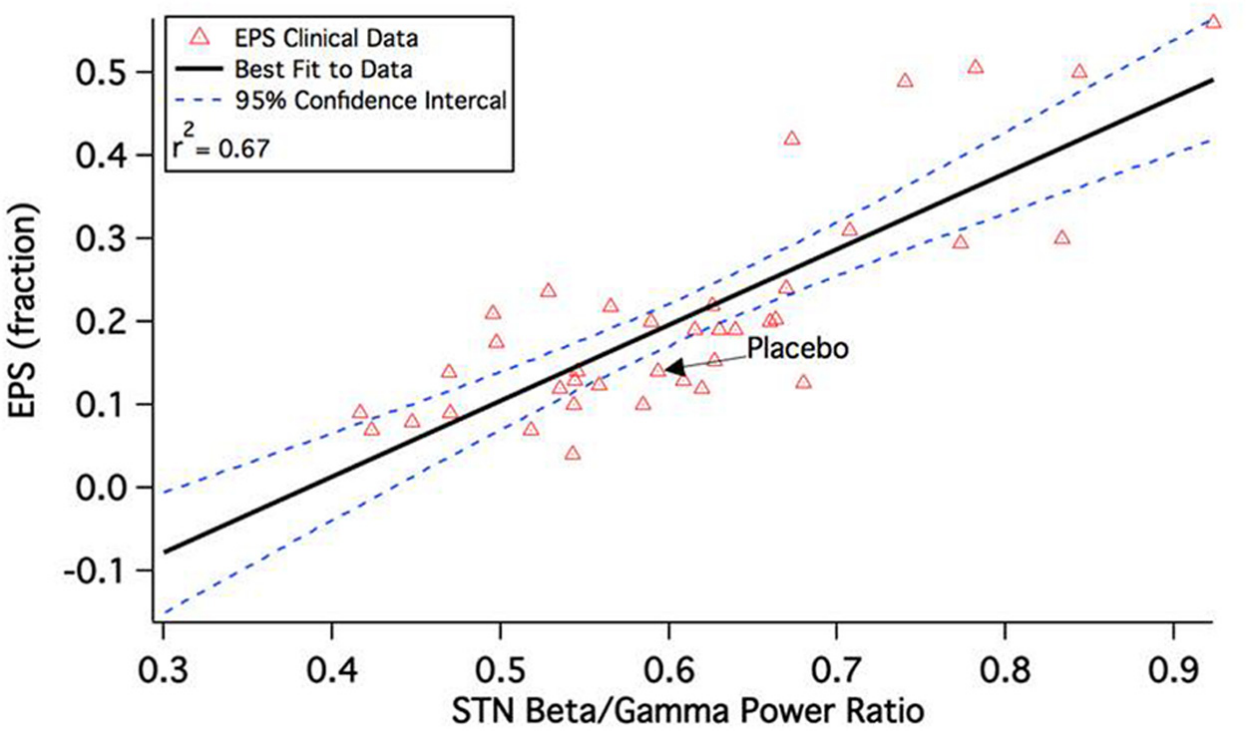
**Correlation between calculated beta/gamma power ratio (STN b/g) for 43 schizophrenia drug-dose combinations in the QSP model and reported extra-pyramidal Parkinsonian motor side-effect liability during a short-term clinical trial.** The clinical readout is the fraction of patients switched to anticholinergic medication at any time during the experimental drug treatment. Note that the correlation is quite substantial and much higher than the correlation between side-effect liability and pure D_2_R antagonism, which is only 0.18 (Spiros et al., 2012).

This leads to a change in the calculated beta/gamma ratio of local field potentials in the STN. **Figure 2** shows the correlation between model outcome and reported Extra-Pyramidal Symptoms (EPS) liability. Adjusting seven parameters (D_1_R effect on D_1_+ MSN via K_ir_, D_2_R effect on D_2_+ MSN via K-A, D_2_R effect on all MSN via AMPA and NMDA max conductances, 5-HT_2A_R effect on Py via HVA and Na_p_ channels, 5-HT_4_R effect on Pyr via K_dr_ and K_ca_ channels, M_1_ mAChR effect on Pyr via K_dr_ channel, and D_1_R effect on Pyr via AMPA and NMDA max conductances from e-e connections) results in a robust correlation between model outcomes and observed clinical readouts. Lower calculated beta/gamma power ratios correspond to lower liabilities of Parkinsonian motor side effects of specific drug-dose combinations.

Interestingly, the EPS liability of atypical antipsychotics that have a substantial 5-HT_2A_R antagonism becomes attenuated at higher doses, while the EPS liability of earlier-generation typical antipsychotics such as Haldol continues to increase monotonically. This is in line with clinical data on the effect of trazodone, suggesting that increasing 5-HT_2A_ antagonisme improves Parkinson’s symptoms (Werneck et al., 2009) and has been demonstrated in an earlier version of the model before (Spiros et al., 2013).

### Implementation of Parkinson’s Pathology

In this section we introduce PD pathology in the QSP model. Neuronal toxicity of dopaminergic neurons in the SN is implemented using decreased DA release levels in the striatal receptor competition model. In addition, we implement both an upregulation of the high-affinity D_2_R and a small compensatory decrease of the DAT transporter, which leads to lower clearance rates and a longer half-life for synaptic dopamine. These changes are based on post-mortem studies in PD patients (Piggott et al., 1999).

**FIGURE 3:**
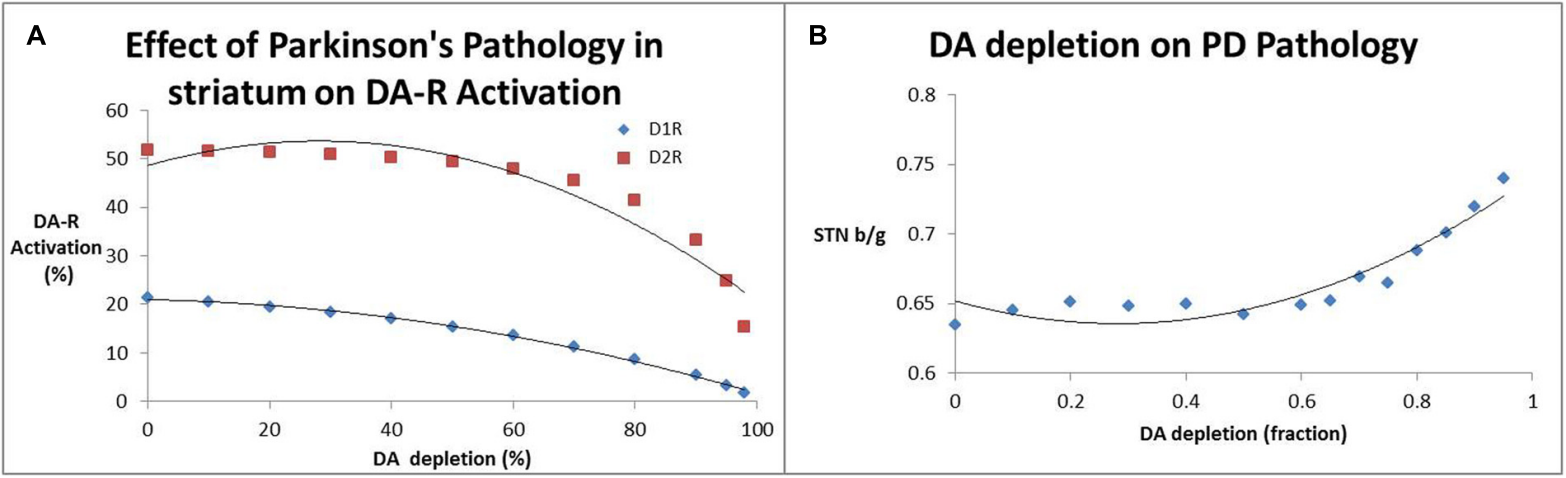
**(A)** Effect of decreased dopamine release (a measure for SN neurotoxicity) on activation of striatal D_1_ and D_2_ receptors. It is clear that substantial decreases of receptor activation only arise at DA depletion levels of 80% and greater. **(B)** Impact of decreased dopamine release (a measure for SN neurotoxicity) on the beta/gamma power ratio (STN b/g) of local field potential oscillations in the subthalamic nucleus. Interestingly the subthalamic nucleus beta/gamma ratio remains relatively unchanged up to a depletion of 60% after which it increases monotonically.

**Figure 3A** shows the effect of decreased presynaptic DA on striatal D_1_ and D_2_R activation levels; substantial declines in receptor activation levels start to become prominent at dopamine depletion levels well beyond 80%. Accordingly, the beta/gamma ratio (**Figure 3B**) only starts to increase substantially for DA depletion above 70%. This non-linear interaction between DA levels, receptor activation and local field potential oscillations might explain some of the clinical observations that symptoms arise only when a large majority of SN neurons have become dysfunctional.

We further tested the differential impact of neuroplasticity at GP_e_-STN coupling compared to changes in MSN-Gpi coupling on local field potentials in STN under Parkinsonian conditions. Supplementary Figure S1 shows that the GP_e_-STN neuroplasticity changes impact beta/gamma readout much less as compared to the MSN-GP_e_ coupling changes. This illustrates the complex relationship between biophysical processes and emergent properties of the network, in this case the oscillations of the local field potential in the STN.

### Clinical Results in Parkinson’s Disease Treatment

#### L-DOPA vs. Partial D2 Agonists

This section deals with the impact of dopaminergic stimulations on the beta/gamma readout. Dopaminergic stimulation can be achieved with L-DOPA, a precursor for dopamine, MAO-B inhibition of neurotransmitter degradation, and dopamine agonists.

The first two cases refer to an increased dopamine release from presynaptic nerve terminals that activate both D_1_ and D_2_R in the direct and indirect pathway.

The actual increase in dopamine release associated with a specific dose of L-DOPA or an MAO-B inhibitor is unknown as no good target engagement data are available. Microdialysis studies in preclinical animal models (Sader-Mazbar et al., 2013) suggest that MAO-B inhibition can easily more than double the level of free ambient dopamine *in vivo*. On the other hand, using RT-132 (a DAT tracer) PET imaging displacement in early PD patients (Guttman et al., 2001), a 22% change was observed after 3 weeks of treatment with 375 mg of L-DOPA corresponding to a doubling of the presynaptic DA release calculated from our receptor competition model. However, in advanced PD, L-DOPA maintenance dose can easily be in the 600–900 mg range. This suggests that MAO-B inhibition is likely to achieve a somewhat smaller increase in free dopamine as compared to L-DOPA with a corresponding smaller clinical benefit.

Many dopamine agonists have been tested in PD. We studied the effect of 12 different compounds, including pramexipole, rotigotine, ropinirole, the D_1_-agonist ABT431, apomorphine and the somewhat older drugs like piribedil, bromocriptine, terguride, cabergoline, lisuride, pergolide, and quinpirole. They interact differently with the dopamine receptors in terms of affinity and maximal agonistic effect. In general, they tend to be more specific for the D_2_S (assumed to be the presynaptic dopamine autoreceptor) than for the D_2_L (post-synaptic dopamine receptor) and have a low affinity for the D_1_R, but interact strongly with the hD_3_R. Notable exceptions are apomorphine (acting on both D_1_ and D_2_R) and ABT431 (specific for the D_1_R). Supplementary Figures S2 and S3 show the pharmacology of the compounds on D_1_, D_2_S, D_2_R, and D_3_ receptors.

**FIGURE 4:**
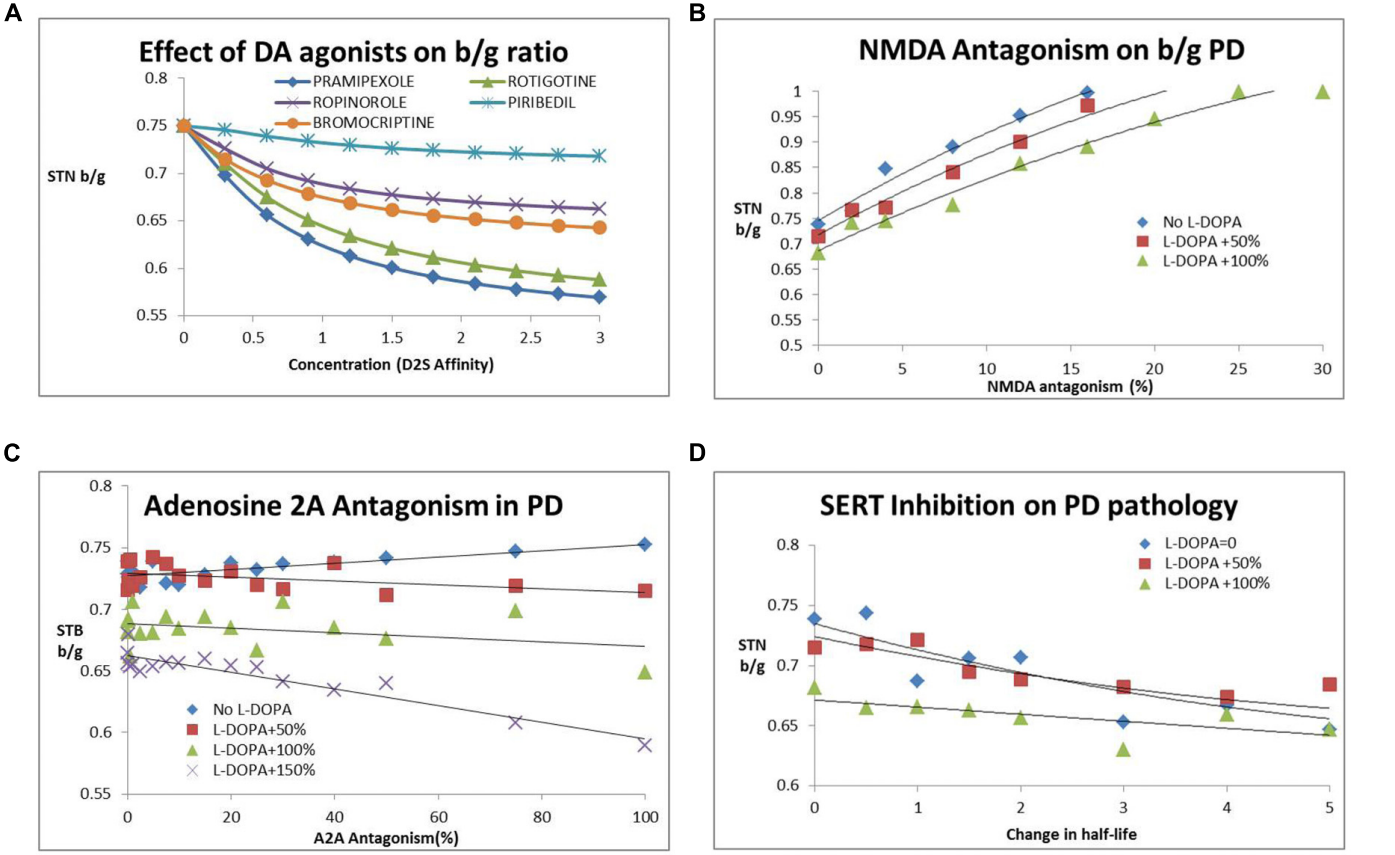
**(A)** Dose-dependent decrease of subthalamic nucleus b/g (STN b/g) with various DA agonists. The simulations suggest that different agonists have different outcomes dependent upon their affinity for pre- and post-synaptic D_2_R with the most recently developed rotigotine and pramepixole having a good robust effect over a range of target engagement levels (expressed as hypothetical D_2_R occupancies) and with minimal liability of dyskinesia at higher doses. **(B)** Effect of NMDA antagonism (NR_2B_ subunit specific) on subthalamic nucleus b/g ratio. Such an approach increases STN beta/gamma ratio (STN b/g) and worsens Parkinsonian symptoms. The number following “L-DOPA” refers to the increase in DA release (normalized to the basal release in the PD condition). The effect is somewhat mitigated by the presence of L-DOPA, with higher L-DOPA comedication levels attentuating the negative effect of NMDA antagonism. Similar effects are observed with an NMDA subtype a-specific inhibitor. **(C)** Adenosine A_2A_R antagonism reduces subthalamic nucleus b/g and improves clinical outcome only in the presence of L-DOPA. The number following “L-DOPA” refers to the increase in DA release (normalized to the basal release in the PD condition). There is no effect or a tendency for increased subthalamic nucleus b/g (and worsened clinical outcome) in the absence of L-DOPA. **(D)** Effect of serotonin transport inhibition and increased 5-HT tone on the subthalamic nucleus b/g ratio as a function of increased 5-HT half-life. The number following “L-DOPA” refers to the increase in DA release (normalized to the basal release in the PD condition). Increased 5-HT tone reduces subthalamic nucleus b/g and improves PD motor symptoms. The effect is already present in the absence of L-DOPA and is mitigated by increasing doses of L-DOPA.

**Figure 4A** shows the effect of different DA agonists on the b/g ratio. The different affinities for D_1_, D_2_S and D_2_L receptors result in different clinical outcomes.

#### Glutamatergic Interventions

A number of glutamatergic interventions have been tested in the clinic, ranging from NMDA antagonism to NR_2B_ specific antagonism and AMPA modulation. The underlying rationale for these targets is based on neuroprotection and the preclinical observations of glutamate overload. Based on studies from the Allen Brain Institute, it was found that the NMDA-NR_2B_ subunit was only present in cortical areas.

After implementation of these biological processes, our computer model predicts an increase in b/g ratio with increasing NMDA antagonism (**Figure 4B**), suggesting a clear worsening of clinical symptoms. For instance a 5% decrease in gNMDA increases b/g between 3% when added to regular L-DOPA dose to 9% as a stand-alone. Subsequent analysis of the effect of a specific NR_2B_ antagonist resulted in a steeper decline of gamma-band power than beta-band power with decreasing conductance of this ligand-gated voltage channel, resulting in an increased beta/gamma power ratio. This suggests that glutamate antagonists in general tend to worsen motor symptoms in the Parkinsonian disease state, in line with observed clinical data on perampanel, MK-0567 and amantadine.

An interesting drug is safinamide, a combination of MAO-B inhibition and NMDA antagonism. These opposing activities tend to balance out; at lower doses the beneficial MAO-B inhibition effect dominates, while at higher doses the negative effect of NMDA antagonism takes over. This is in line with clinical observations (Stocchi et al., 2012) that both the 50 and 100 mg, but not the higher doses of 200 mg improved motor symptoms.

#### Adenosine A2A Intervention

Different inhibitors of the adenosine 2A (A_2A_) receptor pathway have been tested in clinical trials. We implemented this effect as a specific co-regulation of the D_2_R in MSN neurons of the indirect pathway. While A_2A_ activation increases cAMP, corresponding to lowering DA tone on the D_2_R (because of the G_i_ coupling), blocking A2A decreases cAMP and amplifies any effect of D_2_R stimulation, although there is no direct effect on the output of D_1_+ MSN neurons. We implemented the adenosine A_2A_ effect as an increase in the coupling parameter between D_2_R activation and changes in conductance of the A-type K^+^ channel. In line with these ideas, we found that A_2A_ antagonism works better with increasing L-DOPA comedication (**Figure 4C**). The effect, however, is relatively modest.

### Serotonin Modulation

Serotonin neurotransmission impacts basal ganglia network dynamics through both direct and indirect ways. Post-synaptic 5-HT_4_, 5-HT_1B_, and 5-HT_1A_ receptors are located in pallidal brain regions (Varnäs et al., 2003; Chen et al., 2008) and affect excitation and inhibition at STN glutamatergic neurons (Stanford et al., 2005).

5-HT_2A_ affects the pyramidal cells in the motor cortex and plays a role in suppressing tremor, one of the cardinal phenotypes of PD. An earlier QSP model (Spiros et al., 2013) illustrated the impact of 5-HT_2A_ antagonists on the cardinal feature of tremor; when implementing this effect in the current QSP model, 5-HT_2A_ antagonism also decreases beta/gamma ratio in a monotonic fashion. Also, lowering 5-HT tone in general tends to lower b/g ratio, suggesting clinical improvement. This is in line with fluoxetine reducing depressive symptoms, but also its beneficial effect on motor symptoms in a clinical open-label study (Kostic et al., 2012; **Figure 4D**).

### Clinical Calibration

The clinical database collected from publicly available data (see Supplementary information) consists of 41 papers on 24 different drugs. When considering changes in UPDRS Motor Score Part III, we can extract 63 experimental data on drug-dose combinations, including placebo outcomes (34 when limited to 12 weeks studies with 12 placebo studies and 47 when limited to 26 weeks studies). When grouping these data together using weighted averages, we end up with 22 different drug-dose combinations when trial duration was limited to 12 weeks. For the change in OFF time, 41 drug-dose combinations were identified (28 for 12 weeks with 12 placebo effects, 40 for up to 39 weeks). Smaller numbers of drug-dose combinations were found for change in UPDRS II (39), change in UPDRS Total (29) and change in ON time (21).

**FIGURE 5:**
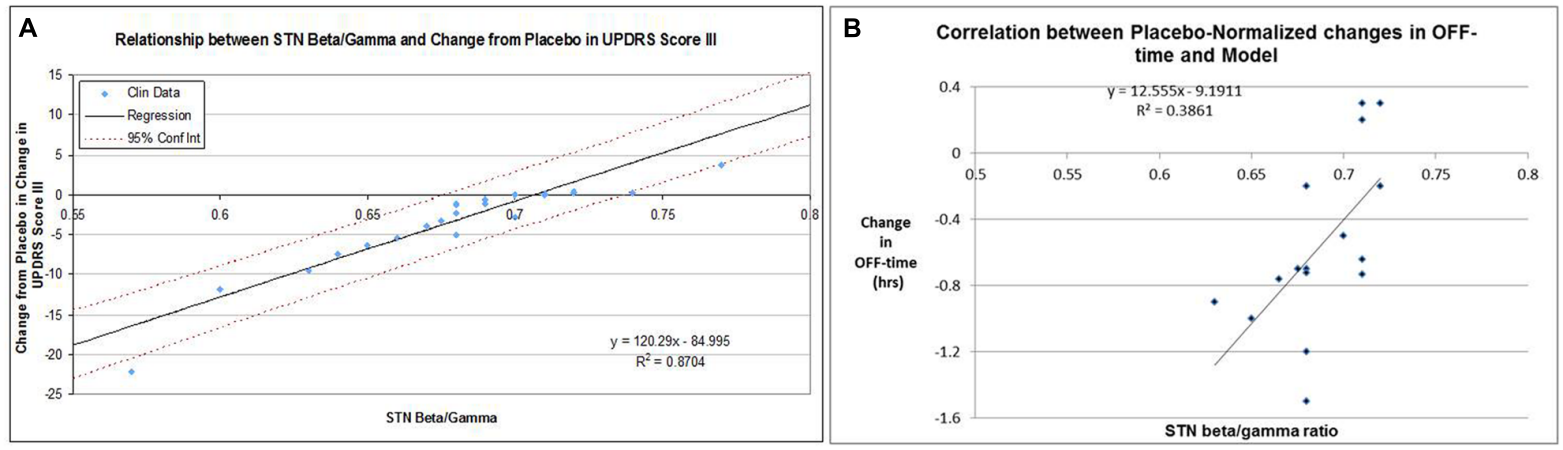
**(A)** Correlation between clinically reported changes between baseline and end-of-treatment (at most 12 weeks) in UPDRS-III score (placebo-controlled) and corresponding QSP model outcomes in calculated subthalamic nucleus b/g (STN b/g). For each data-point the baseline-end-of-treatment difference for the placebo arm of that study was subtracted. In the absence of clear target engagement data, these were optimized within biological ranges with respect for the target engagement of different doses of the same compound. The data suggest that a high correlation can be achieved. **(B)** Correlation between clinically reported changes between baseline and end-of-treatment in OFF-time for 15 drug-dose combinations in short-term studies (12 weeks) and the corresponding QSP model outcomes (subthalamic nucleus b/g ratio), using the optimized parameters for calibration between UPDRS Part III Motor scores and the QSP model. For each data-point the baseline-end-of-treatment difference for the placebo arm of that study was subtracted. The relatively modest correlation (0.386) is also driven by the weak correlation between changes in UPDRS-Part III scores and changes in OFF-time for those studies that reported both outcomes.

Adjusting the target engagement levels within a biological range resulted in a correlation of *R*^2^ = 0.87 for the 22 drug-dose combinations in the 12-weeks studies between QSP model outcomes and clinically observed changes from baseline after correction for the placebo effect (**Figure 5A**). Using these same parameters, we arrived at a correlation of *R*^2^ = 0.39 for the 16 placebo-normalized drug-dose combinations between the clinically reported changes in OFF time and the QSP model outcomes (**Figure 5B**).

### Placebo Effect

Historically, a large placebo effect has been observed in clinical trials in PD. Our database suggests a placebo effect of -9.5 ± 4.5 points on the UPDRS Part III Motor score in an acute setting (four datapoints) and a placebo effect of -1.73 ± 1.23 points for chronic situations (eight datapoints). We hypothesized that this placebo effect was due to a transient dopamine surge. Evidence for this comes from an acute ^11^C-raclopride PET tracer displacement study in healthy volunteers (Boileau et al., 2007; 23% displacement) and a chronic PET displacement study with the dopamine transporter selective tracer ^11^C-RTI32 in Parkinson’s patients (11% displacement; Guttman et al., 2001).

**FIGURE 6:**
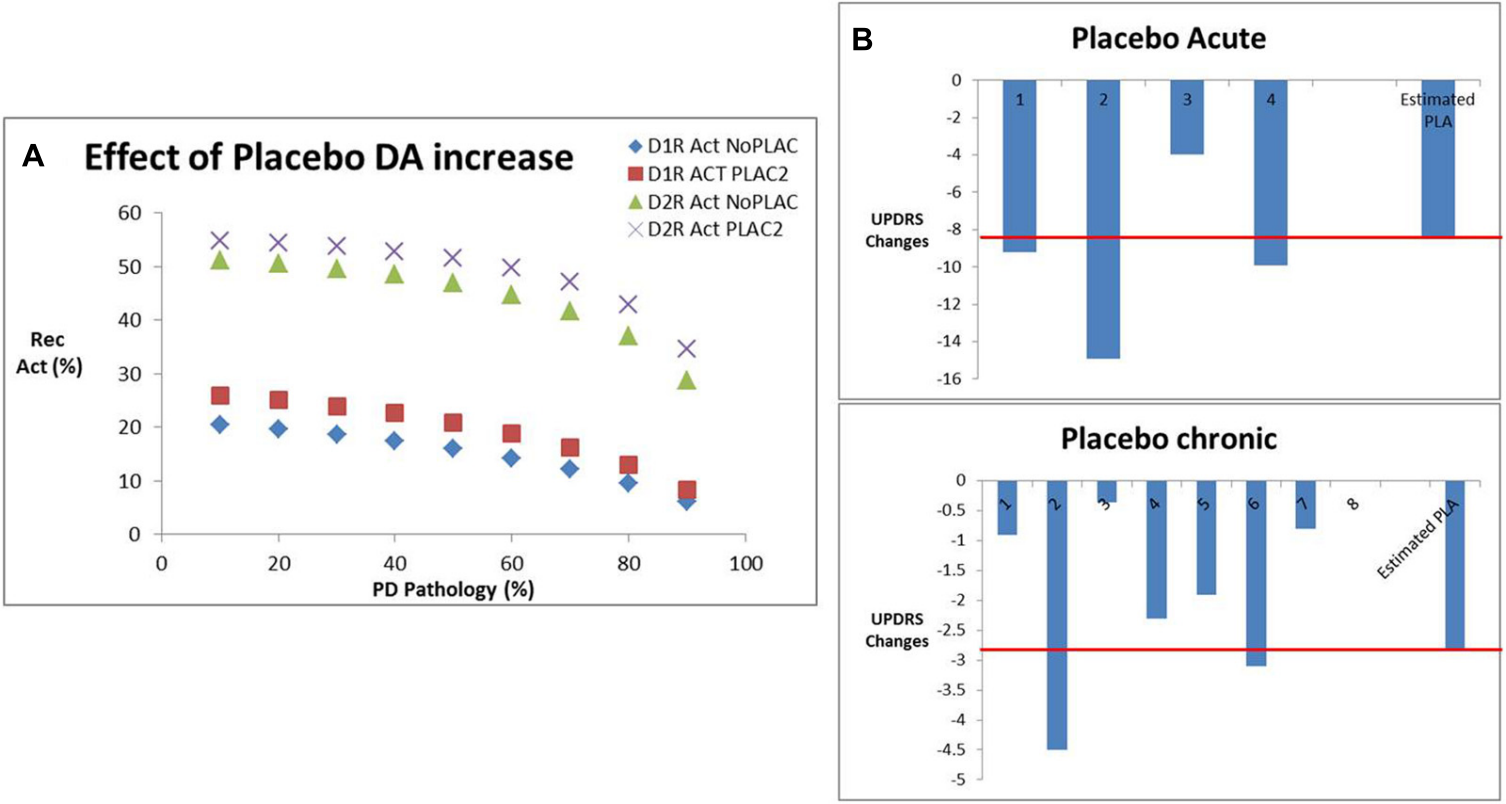
**(A)** Effect of placebo conditions on dopamine increase derived from PET studies in human volunteers and patients with Parkinson’s disease, in particular the impact on D_1_ and D_2_R activation for various levels of dopamine depletion corresponding to Parkinsonian pathology. **(B)** Effect of placebo-induced DA increases from **(A)** on the estimated clinical placebo response derived from our calibrated beta/gamma ratio. Shown are the different clinical studies reporting acute effects of placebo with an average of -7.8 points, where the QSP model predicted an effect of -8.7 points (red line). Similarly, for the chronic placebo case, the average clinical outcome was -2.0, whereas the QSP model predicted an improvement of -2.85 points (red line).

We calculated the increase in dopamine release that would correspond to the clinically observed displacement of both PET tracers using the humanized dopamine receptor competition model (**Figure 6A**). This corresponds to an increase of 120% for the acute situation and 40% for the chronic situation. Using the correlation function determined above, we determined an estimated improvement of 8.7 points on the UPDRS Part III (close to the observed 7.8 points) for the acute placebo effect. Conversely for the chronic situation the model estimated a placebo improvement of 2.85 points which is 39% greater than the observed effect in clinical situations (2.05; **Figure 6B**).

## Discussion

This report presents a QSP implementation of humanized cortico-striatal-thalamo-cortical loop that drives motor behavior. The computer model is based on the neuro-anatomical relationship between different basal ganglia regions (Bazhenov et al., 1998; Rubin and Terman, 2004), i.e., the cortico-striata-thalamo-cortical loop, the nature of the specific cell types, and the GPCRs and both ligand- and voltage-gated ion channels that modulate their respective firing activity. We then implemented the neuropathology and compensatory changes due to Parkinson’s pathology and finally calibrated with clinical data of actual clinical interventions and their impact on UPDRS Part III Motor Score.

With regard to the choice of the model readout, there is ample biological evidence from deep-brain recordings in PD patients that the local field potential power spectrum in the STN, rather than firing frequency in any other region, is directly related to motor symptoms such as bradykinesia and rigidity (Alonso-Frech et al., 2006; Sharott et al., 2014). Interestingly, the low-gamma power spectrum (30–45 Hz) is associated with clinical tremor (Beudel et al., 2015). Therefore we calculate the ratio of beta-power over gamma power of the local field potentials in the STN as an *in silico* biomarker for motor symptoms. This also underscores at the same time the richness of information contained in local field potential oscillations and the extreme difficulty to predict what the impact of a certain intervention at a specific target would be without any computational support.

The model captures the observation that clinical symptoms (i.e., substantial changes in the beta/gamma ratio) become apparent after 70% of dopamine tone is lost. This is partly due to the non-linear interaction at the level of activation of post-synaptic D_1_ and D_2_R when presynaptic dopamine release is reduced.

The results suggest that the platform can achieve a robust correlation with historical clinical trials, including the therapeutic interventions that did not show a clinical benefit. Part of this high correlation with the UPDRS Part III Motor symptoms is driven by the fact that we could adjust target engagement levels for the different drugs for which no actual target exposure data were available (see further below). Using the same target engagement levels the correlation between model outcome and OFF time was smaller, suggesting that these clinical scales report on different clinical phenotypes. Indeed in the small number of studies (*n* = 19) where both data are available for the same patient groups, the correlation between changes in UPDRS Part III Motor symptoms and OFF time was only *R*^2^ = 0.417.

It has to be noted also the biological coupling parameters were all adjusted using the correlation between model outcome in a schizophrenia environment and the reported clinical data on the fraction of patients that needed anticholinergic medication to address their EPS side-effects. No further adjustment was made for the PD state, except for the parameters that defined the neuroplasticity changes at the level of MSN-GP_i_ and GP_e_-STN.

A major issue is the lack of target engagement in the reported clinical trials. This is mainly due to the absence of good radiotracers that can report on D*_2_* agonism. In a small clinical study pramexipole was documented to reduce the binding of FLB457, a specific D*_2_* antagonist radiotracer by 10% at a dose of 0.125 mg and 20% at the dose of 0.25 mg (Ishibashi et al., 2011). In contrast, a study with a specific radiotracer SCH442416 revealed that A*_2A_* antagonists can displace up to 90% (Brooks et al., 2010), corresponding to an increase of D*_2_*R coupling to A-type K^+^ channel of 65%. Where available, we used this target engagement information together with the assumption of linear pharmacokinetics for different doses of the same drug. This allows one to achieve a substantial correlation between historical clinical reported studies and computer model outcomes for the same drug-dose combinations.

The observations with serotonin transport inhibitors suggest a substantial beneficial effect on STN b/g and therefore on clinical symptoms improvement. However, higher doses of SERT inhibitors are associated with severe side-effects including cognitive worsening (Porsteinsson et al., 2014), which can be an issue, especially in elderly PD patients. This limits the usefulness of this therapeutic intervention.

Limitations of the model include the level of resolution in the different basal ganglia subregions. Due to computational constraints we only have a finite number of neurons in each basal ganglia subregion, ranging from 4 to 16. This does not allow us to more finely distinguish between different subregions of each basal ganglia region.

We do not model explicitly the impact of serotonergic synapse derived dopamine with L-DOPA as proposed in earlier computational models (Best et al., 2010; Reed et al., 2012); for L-DOPA treatment we calibrated the response in terms of increase in free DA levels, irrespective of the origin of DA release. We acknowledge that this can be modulated by interventions that change 5-HT tone such as SERT inhibitors or 5-HT*_1A/B_* modulators. However, *in vivo* rat microdialysis studies suggest that this effect might be limited (Paolone et al., 2015).

While the computer model incorporates different neurotransmitter systems, such as dopamine, serotonin, acetylcholine, adrenerge, glutamate and GABA, it falls short on other systems that have been documented to be involved in basal ganglia physiology and PD pathology, such as the endocannabinoid system, reviewed in More and Choi (2015). For instance, affecting this systems with a phytocannabinoid Δ(9)-THCV that stimulates CB_2_ and blocks CB_1_ receptors provide symptomatic relief and neuroprotection in animal models (García et al., 2011). The cannabinoid agonist WIN 55212-2 has been studied extensively in preclinical animal models of PD (Price et al., 2007; Morgese et al., 2009). However, clinical trials to date have not shown an robust and reproducible effect (Kluger et al., 2015), illustrating the complex pharmacology of this neurotransmitter system.

The endocannabinoid system affect dopaminergic signaling possibly through a heterodimerization between CB_1_ and D_2_ (Marcellino et al., 2008) and modulates glutamate (Grundy et al., 2001) and GABA levels (Brotchie, 2003), in addition to direct effects on MSN excitability (Aceves et al., 2011). This might lead to very complex non-linear interactions that could in principle be simulated in the QSP model with regard to the effect on field potential oscillations, especially in the presence of comedications such as L-DOPA or DA agonists. A detailed simulation of the clinical trials with these compounds could also shed some light on why some trials are successful and others fail.

The biology of the human basal ganglia is very complex and this computer model certainly does not include all biological processes that possibly might play a role. Detailed modeling of all the biological processes of complex circuits is still years away, but reconstruction and simulation of a small neocortical circuit has been reported as a first small step (Markram et al., 2015). However, we would argue that the level of granularity and detail in our computer model although incomplete has value for therapeutic applications in the pharmaceutical industry. Animal models might be more complete, but lack translationability to the human clinical situation. In that regard, QSP has a place alongside traditional preclinical animal models in Drug Discovery and Development as it is an independent and more humanized and clinically calibrated, albeit simpler tool. We also posit that such computer modeling platforms will become better and more predictive as they learn from failed clinical trials and ultimately become a actionable Knowledge Repository platform, not unlike the concept of Computer-Aided Design in engineering industries. Obviously the real validation of this approach for PD will be in the blinded prospective prediction of a clinical trial outcome for new untested drug, similar to the case for the QSP prediction of a novel Alzheimer drug (Nicholas et al., 2013) or new untested drugs for schizophrenia (Geerts et al., 2012; Liu et al., 2014).

This simulation platform in its current form also does not address the issue of neurotoxicity and is only intended to study symptomatic effects. In order to minimize possible neuroprotective effects on specific interventions we limited the duration of clinical trials for the calibration to 12 weeks. These and other biological processes like the endocannabinoid systems can in principle be added later into a more comprehensive model.

In summary, this report documents a QSP model of a closed cortico-striatal-thalamocortical loop of the motor striatum that is fully calibrated using human clinical data on therapeutic interventions in PD. The platform could be used to support the development of new symptomatic drugs or to evaluate the impact of various comedications in clinical practice. In principle, this model could also be expanded to simulate the complex emergent properties of other cortico-basal ganglia loops such as the meso-limbic or associative loops and therefore could address other important questions, such as psychiatric symptoms like obsessive behavior, agitation or cognitive aspects.

## Author Contributions

PR developed the model and AS calibrated the model; HG collected clinical information and wrote the paper.

## Conflict of Interest Statement

The authors declare that the research was conducted in the absence of any commercial or financial relationships that could be construed as a potential conflict of interest.

## Abbreviations

b/g: ratio of beta-power to gamma power
CB: cannabinoid system (receptor)
CNS: central nervous systems
COMT: catechol-*O*-methyl transferase
DA: dopamine
GPCR: G-protein coupled receptor
GP_e_: globus pallidus externa
GP_i_: globus pallidus interna
L-DOPA: L-3,4-dihydroxyphenylalanine (precursor for dopamine)
MAO-B: mono-amine oxidase type B
MSN: medium spiny neuron, the dominant GABAerge cell type in striatum
NMDA: *N*-methyl-D-aspartate
PDE-10A: phosphodiesterase 10A
PD: Parkinson’s disease
PET: Positron emission tomography
QSP: Quantitative Systems Pharmacology
RE: reticular GABAergic thalamic interneuron
SN: substantia nigra
STN: subthalamic nucleus
STN b/g: ratio of beta-power over gamma power of the local field potentials in the subthalamic nucleus
TC: thalamocortical projection neurons
