# Supplementary material for: A Humanized Clinically Calibrated Quantitative Systems Pharmacology Model for Hypokinetic Motor Symptoms in Parkinson’s Disease
Source: Front Pharmacol. 2016 Feb 2;7:6. doi: 10.3389/fphar.2016.00006 (PMC4735425; doi:10.3389/fphar.2016.00006)
Supplement: Supplementary file 1 [file Data_Sheet_1.PDF]

SUPPLEMENTARY INFORMATION

A HUMANIZED CLINICALLY CALIBRATED QUANTITATIVE SYSTEMS PHARMACOLOGY MODEL FOR HYPOKINETIC MOTOR SYMPTOMS IN PARKINSON'S DISEASE

Hugo Geerts*, Patrick Roberts, Athan Spiros

Correspondence : Hugo Geerts, hugo-geerts@in-silico-biosciences.com

Supplementary Figures

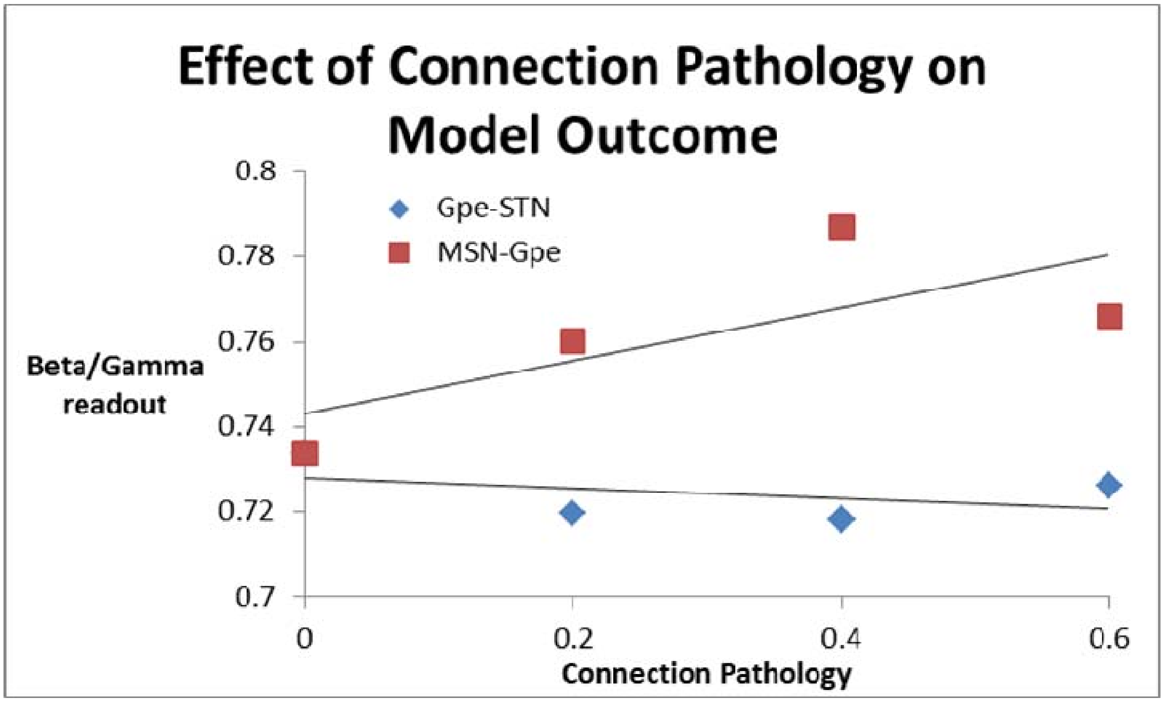

Fig S1: Sensitivity analysis of coupling between MSN and GP_e and between GP_e and STN on beta/gamma ratio in subthalamic nucleus readout. It turns out that the impact of MSN to Gp_e coupling is higher than the impact of GP_e to STN coupling.

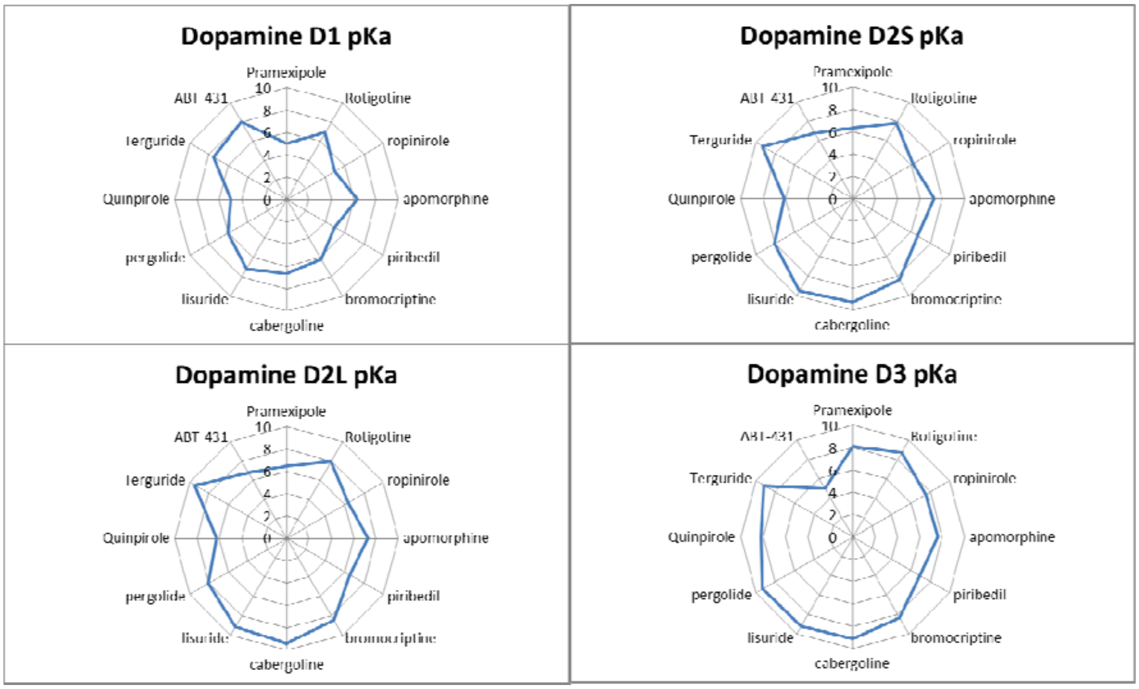

Fig S2. pKa of the different dopamine agonists for hD_1, hD_2S, hD_2L and hD_3 used in calibrating the Parkinsonian QSP model.

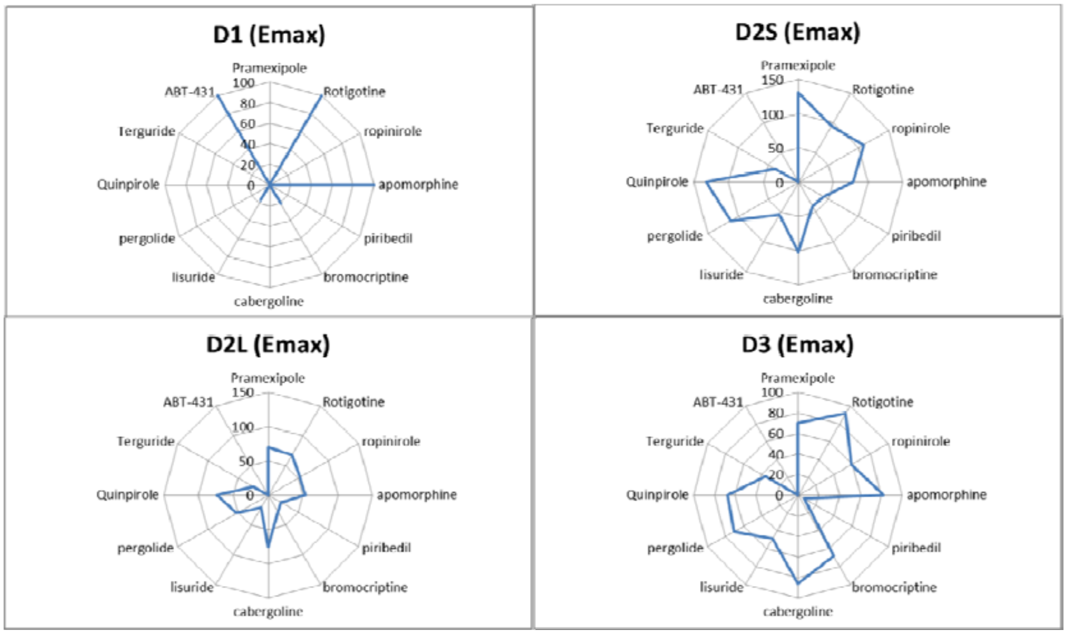

Fig S3. Emax of the different dopamine agonists for hD_1, hD_2S, hD_2L and hD_3 used in calibrating the Parkinsonian QSP model.

Supplementary Tables

Table S1. List of interactions implemented in the Quantitative Systems Pharmacology model

**Receptor**	**Physiological implementation**	**Reference**
Dopamine D_1R in cortex pyramidal cells	Increases NMDA, decreases AMPA and increases GABA conductance	(Law-Tho, Hirsch et al. 1994)
Dopamine D_2R	presynaptic autoreceptor; modulates AMPAR on pyramidal cells	(Tseng and O'Donnell 2007)
Dopamine D_4R	Affects AMPA-R function	(Yuen and Yan 2009)
Serotonin 5-HT_{1A}	Affects Na^+-currents	(Foehring 1996; Cardenas, Del Mar et al. 1997)
Serotonin 5-HT_{1B}	presynaptic autoreceptor in 5HT synapse	(Gothert 1990)
Serotonin 5-HT_{2A}	Affects Na^+ and High Threshold Ca^{++}-currents	(Carr, Cooper et al. 2002)
Serotonin 5-HT_{2C} in striatum	Reduces DA release	(Abdallah, Bonasera et al. 2009)
Serotonin 5-HT_3 in ventral striatum	Stimulates DA release	(De Deurwaerdere, Moison et al. 2005)
Serotonin 5-HT_3	Modulates GABA conductance	(Puig, Santana et al. 2004)
Serotonin 5-HT_4	Affects serotonergic firing rates; affects K_{dr} and K_s and GABA in pyramidal cells	(Ansanay, Dumuis et al. 1995; Cai, Flores-Hernandez et al. 2002)
Serotonin 5-HT_6	Modulates Levels of ACh, NE and DA	(Riemer, Borroni et al. 2003)
Serotonin 5-HT_6 in	Reduces Ventral Tegmentum Area (VTA)	(Minabe, Shirayama

striatum	firing	et al. 2004)
Adrenergic Alpha$_{1A}$ in amygdala	Reduces excitatory afferent input in striatum	(Braga, Aroniadou-Anderjaska et al. 2004)
Adrenergic Alpha$_{2A}$	Modulates K^+ conductance on inhibitory interneurons	(Boehm 1999)
Muscarinic M_1R	Cl leak in MSN & K_{dr} in pyramidal cells	(Perez-Rosello, Figueroa et al. 2005; Shen, Hamilton et al. 2005)
Muscarinic M_2R	Presynaptic autoreceptor for cholinergic synapses;	(Zhang, Yamada et al. 2002; Parnas 2005)
Muscarinic M_2R in striatum	Modulates afferent Glu from cortex	(Calabresi and Di Filippo 2008)
Nicotinic □$_7$ AChR	Increases presynaptic Glu release, increases interneuron excitability	(Alkondon and Albuquerque 2001; Parikh, Ji et al. 2010)
Nicotinic □$_4$□$_□$ AChR in	Increases presynaptic GABA release	(Aracri, Consonni et al. 2010)
$mGluR_2$	Presynaptic Glu autoreceptor; affects Glu release	(Mateo and Porter 2007)

Table 1. List of receptors effects implemented in the model, their localization and physiological effect. All processes are described for the cortex, except where noted.

2. CLINICAL INTERVENTIONS IN SCHIZOPHRENIA : PARKINSONIAN EXTRA-PYRAMIDAL MOTOR SIDE-EFFECTS

Table S2. LIST OF CLINICAL TRIALS USED IN CALIBRATION OF ANTIPSYCHOTIC-INDUCED EPS MOTOR SYMPTOMS

DRUG	CLINICAL TRIAL
Amisulpride	(Delcker, Schoon et al. 1990; Wetzel, Grunder et al. 1998; Sechter, Peuskens et al. 2002; Mortimer, Martin et al. 2004)
Amoxapine	(Fitzgerald, Filia et al. 2004; Apiquian, Fresan et al. 2005; Chaudhry, Husain et al. 2007)
Asenapine	(Kane, Mackle et al. 2011; Szegedi, Verweij et al. 2012)
Aripiprazole	(Kane, Carson et al. 2002; Casey, Carson et al. 2003; Potkin, Saha et al. 2003; Christensen, Poulsen et al. 2006; Tandon, Marcus et al. 2006; Kane, Crandall et al. 2007; Salzman, Rosenberg et al. 2007)
Bifeprunox	(Casey, Sands et al. 2008)
Chlorpromazine	(Claghorn, Honigfeld et al. 1987)
Clozapine	(Claghorn, Honigfeld et al. 1987; Klieser, Lehmann et al. 1995; Brown, Gewirtz et al. 1997; Buchanan, Breier et al. 1998; Breier, Malhotra et al. 1999; Rosenheck, Cramer et al. 1999; Rosenheck, Dunn et al. 1999; Simpson, Josiassen et al. 1999; Citrome, Volavka et al. 2001; Volavka, Czobor et al. 2002; Spivak, Shabash et al. 2003; Naber, Riedel et al. 2005)
Fananserin	(Truffinet, Tamminga et al. 1999)
Flupenthixol	(Wetzel, Grunder et al. 1998; Hertling, Philipp et al. 2003; Gattaz, Diehl et al. 2004)
Fluphenazine	(Chouinard, Annable et al. 1989; Dossenbach, Folnegovic-Smalc et al. 2004)
Haloperidol	(Chouinard, Annable et al. 1989; Fleischhacker, Barnas et al. 1989; Ahlfors, Rimon et al. 1990; Andersen, Korner et al. 1990; Delcker, Schoon et al. 1990; Lapierre, Nair et al. 1990; Claus, Bollen et al. 1992; Ceskova and Svestka 1993; Min, Rhee et al. 1993; Blin, Azorin et al. 1996; Brown, Gewirtz et al. 1997; Tollefson, Beasley et al. 1997; Zimbroff, Kane et al.

	1997; Jeste, Lacro et al. 1999; Tran, Tollefson et al. 1999; Copolov, Link et al. 2000; Heck, Haffmans et al. 2000; Bernardo, Parellada et al. 2001; Cavallaro, Mistretta et al. 2001; Citrome, Volavka et al. 2001; Garcia-Cabeza, Gomez et al. 2001; Gomez and Crawford 2001; Csernansky, Mahmoud et al. 2002; Kane, Carson et al. 2002; Tauscher, Kufferle et al. 2002; Volavka, Czobor et al. 2002; Kennedy, Jeste et al. 2003; Spivak, Shabash et al. 2003; Dossenbach, Erol et al. 2004; Corripio, Catafau et al. 2005; Zipursky, Christensen et al. 2005; Chaudhry, Husain et al. 2007; Kane, Crandall et al. 2007; Potkin, Weiden et al. 2009)
iloperidone	(Potkin, Litman et al. 2008; Cutler, Kalali et al. 2013)
lurasidone	(Nakamura, Ogasa et al. 2009; Loebel, Cucchiaro et al. 2013; McEvoy, Citrome et al. 2013; Stahl, Cucchiaro et al. 2013)
melperone	(Sumiyoshi, Jayathilake et al. 2003; Sumiyoshi, Jayathilake et al. 2003)
methotrimepraz ine	(Blin, Azorin et al. 1996)
Olanzapine	(Bernardo, Parellada et al. 2001; Citrome, Volavka et al. 2001; Garcia-Cabeza, Gomez et al. 2001; Gomez and Crawford 2001; Volavka, Czobor et al. 2002; Gureje, Miles et al. 2003; Kennedy, Jeste et al. 2003; Advokat, Dixon et al. 2004; Ascher-Svanum, Zhu et al. 2004; Dossenbach, Erol et al. 2004; Dossenbach, Folnegovic-Smalc et al. 2004; Gattaz, Diehl et al. 2004; Mortimer, Martin et al. 2004; Cooper, Moisan et al. 2005; Dollfus, Olivier et al. 2005; Zipursky, Christensen et al. 2005)
Paliperidone	(Kramer, Litman et al. 2010; Li, Rui et al. 2011; Alphs, Bossie et al. 2013; Kim, Chang et al. 2013; Na, Kim et al. 2013)
perphenazine	(Lepola, Koskinen et al. 1989)
Quetiapine	(Copolov, Link et al. 2000; Mullen, Jibson et al. 2001; Dossenbach, Erol et al. 2004)
Remoxipride	(Ahlfors, Rimon et al. 1990; Andersen, Korner et al. 1990; Lapierre, Nair et al. 1990; Walinder and Holm 1990; Vartiainen, Leinonen et al. 1993)

Risperidone	(Claus, Bollen et al. 1992; Ceskova and Svestka 1993; Min, Rhee et al. 1993; Chouinard 1995; Huttunen, Piepponen et al. 1995; Klieser, Lehmann et al. 1995; Blin, Azorin et al. 1996; Jeste, Klausner et al. 1997; Marder, Davis et al. 1997; Breier, Malhotra et al. 1999; Jeste, Lacro et al. 1999; Bouchard, Merette et al. 2000; Heck, Haffmans et al. 2000; Cavallaro, Mistretta et al. 2001; Citrome, Volavka et al. 2001; Garcia-Cabeza, Gomez et al. 2001; Mullen, Jibson et al. 2001; Csernansky, Mahmoud et al. 2002; Sechter, Peuskens et al. 2002; Tauscher, Kufferle et al. 2002; Volavka, Czobor et al. 2002; Gureje, Miles et al. 2003; Hertling, Philipp et al. 2003; Potkin, Saha et al. 2003; Advokat, Dixon et al. 2004; Ascher-Svanum, Zhu et al. 2004; Dossenbach, Erol et al. 2004; Apiquian, Fresan et al. 2005; Cooper, Moisan et al. 2005; Dollfus, Olivier et al. 2005; Kim, Shin et al. 2005)
sertindole	(van Kammen, McEvoy et al. 1996; Zimbroff, Kane et al. 1997)
sonepiprazole	(Corrigan, Gallen et al. 2004)
sulpiride	(Lepola, Koskinen et al. 1989)
ziprasidone	(Corripio, Catafau et al. 2005; Potkin, Weiden et al. 2009; Sciarretta and Piazzi 2012)
zotepine	(Dieterle, Ackenheil et al. 1987; Fleischhacker, Barnas et al. 1989; Lin, Chiu et al. 2013)
zuclopenthixol	(Huttunen, Piepponen et al. 1995)

2. CLINICAL INTERVENTIONS IN PARKINSON'S DISEASE

- L-DOPA : IPX066, Carbidopa
- Dopamine agonists: roprinole, rotigotine, apomorphine, pramexipole, piribedil, terguride
- COMT inhibitors : entacapone, tolcapone
- MAO-B inhibitors: selegeline, rasagiline, safinamide
- 5-HT modulators : trazodone, fluoxetine, duloxetine
- A2A modulators : istradefylline, caffeine, preladenant
- NMDA antagonists : amantadine, memantine, flupirtine, MK-0657
- Ca-channel blockers : isradipine
- AMPA antagonist : perampanel

Table S2. LIST OF CLINICAL TRIALS USED IN CALIBRATION OF PARKINSON'S DISEASE

**Drug**	**Major pharmacological activity**	**Reference**
Perampanel	AMPA-R antagonist	(Eggert, Squillacote et al. 2010; Lees, Fahn et al. 2012; Rascol, Barone et al. 2012)
Entacapone	COMT inhibitor	(Rascol, Barone et al. 2012)
MK-0657	NR2B antagonist	(Addy, Assaid et al. 2009)
Carbidopa/L-Dopa	Dopamine precursor	(Addy, Assaid et al. 2009)
Memantine	NMDA antagonist	(Moreau, Delval et al. 2013)
Flupertine	NMDA antagonist	(Putzki, Maschke et al. 2002)
Istradefylline	Adenosine A2A antagonist	(Hauser, Shulman et al. 2008; LeWitt, Guttman et al. 2008; Stacy, Silver et al. 2008; Fernandez, Greeley et al. 2010; Mizuno, Hasegawa et al. 2010; Pourcher, Fernandez et al. 2012; Mizuno and Kondo 2013)
Caffeine	Adenosine A2A antagonist	(Wills, Eberly et al. 2013)
Preladenant	Adenosine A2A antagonist	(Hauser, Cantillon et al. 2011; Factor, Wolski et al. 2013)
Pramipexole	DA agonist	(Hauser, Schapira et al. 2014; Wang, Sun et al. 2014)

Rasagiline	MAO-B inhibitor	(Hauser, Silver et al. 2014; Jankovic, Berkovich et al. 2014)
isradipine	Ca-channel blocker	(2013)
apomorphine	DA analog	(Pahwa, Koller et al. 2007; Hattori and Nomoto 2014)
Apomorphine VR040	DA analog	(Grosset, Malek et al. 2013; Grosset, Malek et al. 2013; Grosset, Malek et al. 2013)
Rotigotine	DA agonist	(LeWitt, Boroojerdi et al. 2013; Giladi, Ghys et al. 2014; Mizuno, Nomoto et al. 2014)
ropinirole	DA agonist	(Mizuno, Nomoto et al. 2014)
safinamide	MAO-B inhibitor & NMDA antagonist	(Stocchi, Borgohain et al. 2012; Borgohain, Szasz et al. 2014; Borgohain, Szasz et al. 2014)
Fluoxetine	SERT inhibitor	(Kostic, Dzoljic et al. 2012; Factor, Wolski et al. 2013)
Trazodone	5-HT modulator	(Werneck, Rosso et al. 2009)
amantadine	NMDA antagonist	(Kim, Yun et al. 2012)
Duloxetine	NET inhibitor	(Bonuccelli, Meco et al. 2012)
tolcapone	COMT inhibitor	(Eggert, Oertel et al. 2014)
IPX066	NMDA antagonist	(Stocchi, Hsu et al. 2014)
opicapone	COMT inhibitor	(Ferreira, Rocha et al. 2015)
Terguride	DA agonist	(Martignoni, Pacchetti et al. 1995; Martignoni, Horowski et al. 1996)
Piribedil	DA agonist	(Ziegler, Castro-Caldas et al. 2003; Suwantamee, Nidhinandana et al. 2004; Castro-Caldas, Delwaide et al. 2006)
Bromocriptine	Cholinergic antagonist	(Castro-Caldas, Delwaide et al. 2006)
ABT-431	D1-agonist	(Rascol, Blin et al. 1999; Rascol, Nutt et al. 2001)
Gabapentin	GABA modulator	(Van Blercom, Lasa et al. 2004)
zonisamide	Na-channel antagonist	(Murata, Hasegawa et al. 2007)
Pergolide	DA agonist	(Storch, Trenkwalder et al. 2005)
Clozapine	antipsychotic	(Morgante, Epifanio et al. 2004)
Quetiapine	antipsychotic	(Morgante, Epifanio et al. 2004)
Methylphenidate	DAT blocker	(Moreau, Delval et al. 2012)

(2013). "Phase II safety, tolerability, and dose selection study of isradipine as a potential disease-modifying intervention in early Parkinson's disease (STEADY-PD)." Mov Disord **28**(13): 1823-1831.

Abdallah, L., S. J. Bonasera, et al. (2009). "Impact of Serotonin 2C Receptor Null Mutation on Physiology and Behavior Associated with Nigrostriatal Dopamine Pathway Function." Journal of Neuroscience **29**(25): 8156-8165.

Addy, C., C. Assaid, et al. (2009). "Single-dose administration of MK-0657, an NR2B-selective NMDA antagonist, does not result in clinically meaningful improvement in motor function in patients with moderate Parkinson's disease." J Clin Pharmacol **49**(7): 856-864.

Advokat, C., D. Dixon, et al. (2004). "Comparison of risperidone and olanzapine as used under "real-world" conditions in a state psychiatric hospital." Prog Neuropsychopharmacol Biol Psychiatry **28**(3): 487-495.

Ahlfors, U. G., R. Rimon, et al. (1990). "Remoxipride and haloperidol in schizophrenia: a double-blind multicentre study." Acta Psychiatr Scand Suppl **358**: 99-103.

Alkondon, M. and E. X. Albuquerque (2001). "Nicotinic acetylcholine receptor alpha7 and alpha4beta2 subtypes differentially control GABAergic input to CA1 neurons in rat hippocampus." J Neurophysiol **86**(6): 3043-3055.

Alphs, L., C. A. Bossie, et al. (2013). "Paliperidone palmitate and risperidone long-acting injectable in subjects with schizophrenia recently treated with oral risperidone or other oral antipsychotics." Neuropsychiatr Dis Treat **9**: 341-350.

Andersen, J., A. Korner, et al. (1990). "A double blind comparative multicentre study of remoxipride and haloperidol in schizophrenia." Acta Psychiatr Scand Suppl **358**: 104-107.

Ansanay, H., A. Dumuis, et al. (1995). "cAMP-dependent, long-lasting inhibition of a K+ current in mammalian neurons." Proc Natl Acad Sci U S A **92**(14): 6635-6639.

Apiquian, R., A. Fresan, et al. (2005). "Amoxapine as an atypical antipsychotic: a comparative study vs risperidone." Neuropsychopharmacology **30**(12): 2236-2244.

Aracri, P., S. Consonni, et al. (2010). "Tonic modulation of GABA release by nicotinic acetylcholine receptors in layer V of the murine prefrontal cortex." Cereb Cortex **20**(7): 1539-1555.

Ascher-Svanum, H., B. Zhu, et al. (2004). "A comparison of olanzapine and risperidone on the risk of psychiatric hospitalization in the naturalistic treatment of patients with schizophrenia." Ann Gen Hosp Psychiatry **3**(1): 11.

Bernardo, M., E. Parellada, et al. (2001). "Double-blind olanzapine vs. haloperidol D2 dopamine receptor blockade in schizophrenic patients: a baseline-endpoint." Psychiatry Res **107**(2): 87-97.

Blin, O., J. M. Azorin, et al. (1996). "Antipsychotic and anxiolytic properties of risperidone, haloperidol, and methotrimeprazine in schizophrenic patients." J Clin Psychopharmacol **16**(1): 38-44.

Boehm, S. (1999). "Presynaptic alpha2-adrenoceptors control excitatory, but not inhibitory, transmission at rat hippocampal synapses." J Physiol **519 Pt 2**: 439-449.

Bonuccelli, U., G. Meco, et al. (2012). "A non-comparative assessment of tolerability and efficacy of duloxetine in the treatment of depressed patients with Parkinson's disease." Expert Opin Pharmacother **13**(16): 2269-2280.

Borgohain, R., J. Szasz, et al. (2014). "Randomized trial of safinamide add-on to levodopa in Parkinson's disease with motor fluctuations." Mov Disord **29**(2): 229-237.

Borgohain, R., J. Szasz, et al. (2014). "Two-year, randomized, controlled study of safinamide as add-on to levodopa in mid to late Parkinson's disease." Mov Disord **29**(10): 1273-1280.

Bouchard, R. H., C. Merette, et al. (2000). "Longitudinal comparative study of risperidone and conventional neuroleptics for treating patients with schizophrenia. The Quebec Schizophrenia Study Group." J Clin Psychopharmacol **20**(3): 295-304.

Braga, M. F., V. Aroniadou-Anderjaska, et al. (2004). "Stress impairs alpha(1A) adrenoceptor-mediated noradrenergic facilitation of GABAergic transmission in the basolateral amygdala." Neuropsychopharmacology **29**(1): 45-58.

Breier, A. F., A. K. Malhotra, et al. (1999). "Clozapine and risperidone in chronic schizophrenia: effects on symptoms, parkinsonian side effects, and neuroendocrine response." Am J Psychiatry **156**(2): 294-298.

Brown, A. S., G. Gewirtz, et al. (1997). "Effects of clozapine on plasma catecholamines and relation to treatment response in schizophrenia: a within-subject comparison with haloperidol." Neuropsychopharmacology **17**(5): 317-325.

Buchanan, R. W., A. Breier, et al. (1998). "Positive and negative symptom response to clozapine in schizophrenic patients with and without the deficit syndrome." Am J Psychiatry **155**(6): 751-760.

Cai, X., J. Flores-Hernandez, et al. (2002). "Activity-dependent bidirectional regulation of GABA(A) receptor channels by the 5-HT(4) receptor-mediated signalling in rat prefrontal cortical pyramidal neurons." J Physiol **540**(Pt 3): 743-759.

Calabresi, P. and M. Di Filippo (2008). "ACh/dopamine crosstalk in motor control and reward: a crucial role for alpha 6-containing nicotinic receptors?" Neuron **60**(1): 4-7.

Cardenas, C. G., L. P. Del Mar, et al. (1997). "Two parallel signaling pathways couple 5HT1A receptors to N- and L-type calcium channels in C-like rat dorsal root ganglion cells." J Neurophysiol **77**(6): 3284-3296.

Carr, D. B., D. C. Cooper, et al. (2002). "Serotonin receptor activation inhibits sodium current and dendritic excitability in prefrontal cortex via a protein kinase C-dependent mechanism." J Neurosci **22**(16): 6846-6855.

Casey, D. E., W. H. Carson, et al. (2003). "Switching patients to aripiprazole from other antipsychotic agents: a multicenter randomized study." Psychopharmacology (Berl) **166**(4): 391-399.

Casey, D. E., E. E. Sands, et al. (2008). "Efficacy and safety of bifeprunox in patients with an acute exacerbation of schizophrenia: results from a randomized, double-blind, placebo-controlled, multicenter, dose-finding study." Psychopharmacology **200**(3): 317-331.

Castro-Caldas, A., P. Delwaide, et al. (2006). "The Parkinson-Control study: a 1-year randomized, double-blind trial comparing piribedil (150 mg/day) with bromocriptine (25 mg/day) in early combination with levodopa in Parkinson's disease." Mov Disord **21**(4): 500-509.

Cavallaro, R., P. Mistretta, et al. (2001). "Differential efficacy of risperidone versus haloperidol in psychopathological subtypes of subchronic schizophrenia." Hum Psychopharmacol **16**(6): 439-448.

Ceskova, E. and J. Svestka (1993). "Double-blind comparison of risperidone and haloperidol in schizophrenic and schizoaffective psychoses." Pharmacopsychiatry **26**(4): 121-124.

Chaudhry, I. B., N. Husain, et al. (2007). "Amoxapine as an antipsychotic: comparative study versus haloperidol." J Clin Psychopharmacol **27**(6): 575-581.

Chouinard, G. (1995). "Effects of risperidone in tardive dyskinesia: an analysis of the Canadian multicenter risperidone study." J Clin Psychopharmacol **15**(1 Suppl 1): 36S-44S.

Chouinard, G., L. Annable, et al. (1989). "A randomized clinical trial of haloperidol decanoate and fluphenazine decanoate in the outpatient treatment of schizophrenia." J Clin Psychopharmacol **9**(4): 247-253.

Christensen, A. F., J. Poulsen, et al. (2006). "Patients with schizophrenia treated with aripiprazole, a multicentre naturalistic study." Acta Psychiatr Scand **113**(2): 148-153.

Citrome, L., J. Volavka, et al. (2001). "Effects of clozapine, olanzapine, risperidone, and haloperidol on hostility among patients with schizophrenia." Psychiatr Serv **52**(11): 1510-1514.

Claghorn, J., G. Honigfeld, et al. (1987). "The risks and benefits of clozapine versus chlorpromazine." J Clin Psychopharmacol **7**(6): 377-384.

Claus, A., J. Bollen, et al. (1992). "Risperidone versus haloperidol in the treatment of chronic schizophrenic inpatients: a multicentre double-blind comparative study." Acta Psychiatr Scand **85**(4): 295-305.
Cooper, D., J. Moisan, et al. (2005). "Ambulatory use of olanzapine and risperidone: a population-based study on persistence and the use of concomitant therapy in the treatment of schizophrenia." Can J Psychiatry **50**(14): 901-908.
Copolov, D. L., C. G. Link, et al. (2000). "A multicentre, double-blind, randomized comparison of quetiapine (ICI 204,636, 'Seroquel') and haloperidol in schizophrenia." Psychol Med **30**(1): 95-105.
Corrigan, M. H., C. C. Gallen, et al. (2004). "Effectiveness of the selective D4 antagonist sonepiprazole in schizophrenia: a placebo-controlled trial." Biol Psychiatry **55**(5): 445-451.
Corripio, I., A. M. Catafau, et al. (2005). "Striatal dopaminergic D2 receptor occupancy and clinical efficacy in psychosis exacerbation: a 123I-IBZM study with ziprasidone and haloperidol." Prog Neuropsychopharmacol Biol Psychiatry **29**(1): 91-96.
Csernansky, J. G., R. Mahmoud, et al. (2002). "A comparison of risperidone and haloperidol for the prevention of relapse in patients with schizophrenia." N Engl J Med **346**(1): 16-22.
Cutler, A. J., A. H. Kalali, et al. (2013). "Long-term safety and tolerability of iloperidone: results from a 25-week, open-label extension trial." CNS Spectr **18**(1): 43-54.
De Deurwaerdere, P., D. Moison, et al. (2005). "Regionally and functionally distinct serotonin3 receptors control in vivo dopamine outflow in the rat nucleus accumbens." J Neurochem **94**(1): 140-149.
Delcker, A., M. L. Schoon, et al. (1990). "Amisulpride versus haloperidol in treatment of schizophrenic patients--results of a double-blind study." Pharmacopsychiatry **23**(3): 125-130.
Dieterle, D. M., M. Ackenheil, et al. (1987). "Zotepine, a neuroleptic drug with a bipolar therapeutic profile." Pharmacopsychiatry **20**(1 Spec No): 52-57.
Dollfus, S., V. Olivier, et al. (2005). "Olanzapine versus risperidone in the treatment of post-psychotic depression in schizophrenic patients." Schizophr Res **78**(2-3): 157-159.
Dossenbach, M., A. Erol, et al. (2004). "Effectiveness of antipsychotic treatments for schizophrenia: interim 6-month analysis from a prospective observational study (IC-SOHO) comparing olanzapine, quetiapine, risperidone, and haloperidol." J Clin Psychiatry **65**(3): 312-321.
Dossenbach, M. R., V. Folnegovic-Smalc, et al. (2004). "Double-blind, randomized comparison of olanzapine versus fluphenazine in the long-term treatment of schizophrenia." Prog Neuropsychopharmacol Biol Psychiatry **28**(2): 311-318.
Eggert, K., W. H. Oertel, et al. (2014). "Safety and efficacy of tolcapone in the long-term use in Parkinson disease: an observational study." Clin Neuropharmacol **37**(1): 1-5.
Eggert, K., D. Squillacote, et al. (2010). "Safety and efficacy of perampanel in advanced Parkinson's disease: a randomized, placebo-controlled study." Mov Disord **25**(7): 896-905.
Factor, S. A., K. Wolski, et al. (2013). "Long-term safety and efficacy of preladenant in subjects with fluctuating Parkinson's disease." Mov Disord **28**(6): 817-820.
Fernandez, H. H., D. R. Greeley, et al. (2010). "Istradefylline as monotherapy for Parkinson disease: results of the 6002-US-051 trial." Parkinsonism Relat Disord **16**(1): 16-20.
Ferreira, J. J., J. F. Rocha, et al. (2015). "Effect of opicapone on levodopa pharmacokinetics, catechol-O-methyltransferase activity and motor fluctuations in patients with Parkinson's disease." Eur J Neurol **22**(5): 815-825, e856.
Fitzgerald, P. B., S. Filia, et al. (2004). "Amoxapine in schizophrenia: a negative double-blind controlled trial." J Clin Psychopharmacol **24**(4): 448-450.
Fleischhacker, W. W., C. Barnas, et al. (1989). "Zotepine vs. haloperidol in paranoid schizophrenia: a double-blind trial." Psychopharmacol Bull **25**(1): 97-100.

Foehring, R. C. (1996). "Serotonin modulates N- and P-type calcium currents in neocortical pyramidal neurons via a membrane-delimited pathway." J Neurophysiol **75**(2): 648-659.
Garcia-Cabeza, I., J. C. Gomez, et al. (2001). "Subjective response to antipsychotic treatment and compliance in schizophrenia. A naturalistic study comparing olanzapine, risperidone and haloperidol (EFESO Study)." BMC Psychiatry **1**: 7.
Gattaz, W. F., A. Diehl, et al. (2004). "Olanzapine versus flupenthixol in the treatment of inpatients with schizophrenia: a randomized double-blind trial." Pharmacopsychiatry **37**(6): 279-285.
Giladi, N., L. Ghys, et al. (2014). "Effects of long-term treatment with rotigotine transdermal system on dyskinesia in patients with early-stage Parkinson's disease." Parkinsonism Relat Disord **20**(12): 1345-1351.
Gomez, J. C. and A. M. Crawford (2001). "Superior efficacy of olanzapine over haloperidol: analysis of patients with schizophrenia from a multicenter international trial." J Clin Psychiatry **62 Suppl 2**: 6-11.
Gothert, M. (1990). "Presynaptic serotonin receptors in the central nervous system." Ann N Y Acad Sci **604**: 102-112.
Grosset, K. A., N. Malek, et al. (2013). "Inhaled apomorphine in patients with 'on-off' fluctuations: a randomized, double-blind, placebo-controlled, clinic and home based, parallel-group study." J Parkinsons Dis **3**(1): 31-37.
Grosset, K. A., N. Malek, et al. (2013). "Inhaled dry powder apomorphine (VR040) for 'off ' periods in Parkinson's disease: an in-clinic double-blind dose ranging study." Acta Neurol Scand **128**(3): 166-171.
Grosset, K. A., N. Malek, et al. (2013). "Phase IIa randomized double-blind, placebo-controlled study of inhaled apomorphine as acute challenge for rescuing 'off' periods in patients with established Parkinson's disease." Eur J Neurol **20**(11): 1445-1450.
Gureje, O., W. Miles, et al. (2003). "Olanzapine vs risperidone in the management of schizophrenia: a randomized double-blind trial in Australia and New Zealand." Schizophr Res **61**(2-3): 303-314.
Hattori, N. and M. Nomoto (2014). "Sustained efficacy of apomorphine in Japanese patients with advanced Parkinson's disease." Parkinsonism Relat Disord **20**(8): 819-823.
Hauser, R. A., M. Cantillon, et al. (2011). "Preladenant in patients with Parkinson's disease and motor fluctuations: a phase 2, double-blind, randomised trial." Lancet Neurol **10**(3): 221-229.
Hauser, R. A., A. H. Schapira, et al. (2014). "Long-term safety and sustained efficacy of extended-release pramipexole in early and advanced Parkinson's disease." Eur J Neurol **21**(5): 736-743.
Hauser, R. A., L. M. Shulman, et al. (2008). "Study of istradefylline in patients with Parkinson's disease on levodopa with motor fluctuations." Mov Disord **23**(15): 2177-2185.
Hauser, R. A., D. Silver, et al. (2014). "Randomized, controlled trial of rasagiline as an add-on to dopamine agonists in Parkinson's disease." Mov Disord **29**(8): 1028-1034.
Heck, A. H., P. M. Haffmans, et al. (2000). "Risperidone versus haloperidol in psychotic patients with disturbing neuroleptic-induced extrapyramidal symptoms: a double-blind, multi-center trial." Schizophr Res **46**(2-3): 97-105.
Hertling, I., M. Philipp, et al. (2003). "Flupenthixol versus risperidone: subjective quality of life as an important factor for compliance in chronic schizophrenic patients." Neuropsychobiology **47**(1): 37-46.
Huttunen, M. O., T. Piepponen, et al. (1995). "Risperidone versus zuclopenthixol in the treatment of acute schizophrenic episodes: a double-blind parallel-group trial." Acta Psychiatr Scand **91**(4): 271-277.
Jankovic, J., E. Berkovich, et al. (2014). "Symptomatic efficacy of rasagiline monotherapy in early Parkinson's disease: post-hoc analyses from the ADAGIO trial." Parkinsonism Relat Disord **20**(6): 640-643.

Jeste, D. V., M. Klausner, et al. (1997). "A clinical evaluation of risperidone in the treatment of schizophrenia: a 10-week, open-label, multicenter trial. ARCS Study Group. Assessment of Risperdal in a Clinical Setting." Psychopharmacology (Berl) **131**(3): 239-247.

Jeste, D. V., J. P. Lacro, et al. (1999). "Lower incidence of tardive dyskinesia with risperidone compared with haloperidol in older patients." J Am Geriatr Soc **47**(6): 716-719.

Kane, J. M., W. H. Carson, et al. (2002). "Efficacy and safety of aripiprazole and haloperidol versus placebo in patients with schizophrenia and schizoaffective disorder." J Clin Psychiatry **63**(9): 763-771.

Kane, J. M., D. T. Crandall, et al. (2007). "Symptomatic remission in schizophrenia patients treated with aripiprazole or haloperidol for up to 52 weeks." Schizophr Res **95**(1-3): 143-150.

Kane, J. M., M. Mackle, et al. (2011). "A randomized placebo-controlled trial of asenapine for the prevention of relapse of schizophrenia after long-term treatment." J Clin Psychiatry **72**(3): 349-355.

Kennedy, J. S., D. Jeste, et al. (2003). "Olanzapine vs haloperidol in geriatric schizophrenia: analysis of data from a double-blind controlled trial." Int J Geriatr Psychiatry **18**(11): 1013-1020.

Kim, C. Y., Y. W. Shin, et al. (2005). "Risperidone dosing pattern and clinical outcome in psychosis: an analysis of 1713 cases." J Clin Psychiatry **66**(7): 887-893.

Kim, E. Y., S. M. Chang, et al. (2013). "Long-term effectiveness of flexibly dosed paliperidone extended-release: comparison among patients with schizophrenia switching from risperidone and other antipsychotic agents." Curr Med Res Opin **29**(10): 1231-1240.

Kim, Y. E., J. Y. Yun, et al. (2012). "Intravenous amantadine for freezing of gait resistant to dopaminergic therapy: a randomized, double-blind, placebo-controlled, cross-over clinical trial." PLoS ONE **7**(11): e48890.

Klieser, E., E. Lehmann, et al. (1995). "Randomized, double-blind, controlled trial of risperidone versus clozapine in patients with chronic schizophrenia." J Clin Psychopharmacol **15**(1 Suppl 1): 45S-51S.

Kostic, V., E. Dzoljic, et al. (2012). "Fluoxetine does not impair motor function in patients with Parkinson's disease: correlation between mood and motor functions with plasma concentrations of fluoxetine/norfluoxetine." Vojnosanit Pregl **69**(12): 1067-1075.

Kramer, M., R. Litman, et al. (2010). "Paliperidone palmitate, a potential long-acting treatment for patients with schizophrenia. Results of a randomized, double-blind, placebo-controlled efficacy and safety study." Int J Neuropsychopharmacol **13**(5): 635-647.

Lapierre, Y. D., N. P. Nair, et al. (1990). "A controlled dose-ranging study of remoxipride and haloperidol in schizophrenia--a Canadian multicentre trial." Acta Psychiatr Scand Suppl **358**: 72-77.

Law-Tho, D., J. C. Hirsch, et al. (1994). "Dopamine modulation of synaptic transmission in rat prefrontal cortex: an in vitro electrophysiological study." Neurosci Res **21**(2): 151-160.

Lees, A., S. Fahn, et al. (2012). "Perampanel, an AMPA antagonist, found to have no benefit in reducing "off" time in Parkinson's disease." Mov Disord **27**(2): 284-288.

Lepola, U., T. Koskinen, et al. (1989). "Sulpiride and perphenazine in schizophrenia. A double-blind clinical trial." Acta Psychiatr Scand **80**(1): 92-96.

LeWitt, P. A., B. Boroojerdi, et al. (2013). "Rotigotine transdermal system for long-term treatment of patients with advanced Parkinson's disease: results of two open-label extension studies, CLEOPATRA-PD and PREFER." J Neural Transm **120**(7): 1069-1081.

LeWitt, P. A., M. Guttman, et al. (2008). "Adenosine A2A receptor antagonist istradefylline (KW-6002) reduces "off" time in Parkinson's disease: a double-blind, randomized, multicenter clinical trial (6002-US-005)." Ann Neurol **63**(3): 295-302.

Li, H., Q. Rui, et al. (2011). "A comparative study of paliperidone palmitate and risperidone long-acting injectable therapy in schizophrenia." Prog Neuropsychopharmacol Biol Psychiatry **35**(4): 1002-1008.

Lin, C. C., H. J. Chiu, et al. (2013). "Switching from clozapine to zotepine in patients with schizophrenia: a 12-week prospective, randomized, rater blind, and parallel study." J Clin Psychopharmacol **33**(2): 211-214.

Loebel, A., J. Cucchiaro, et al. (2013). "Efficacy and safety of lurasidone 80 mg/day and 160 mg/day in the treatment of schizophrenia: a randomized, double-blind, placebo- and active-controlled trial." Schizophr Res **145**(1-3): 101-109.

Marder, S. R., J. M. Davis, et al. (1997). "The effects of risperidone on the five dimensions of schizophrenia derived by factor analysis: combined results of the North American trials." J Clin Psychiatry **58**(12): 538-546.

Martignoni, E., R. Horowski, et al. (1996). "Effects of terguride on anterior pituitary function in parkinsonian patients treated with L-dopa: a double-blind study versus placebo." Clin Neuropharmacol **19**(1): 72-80.

Martignoni, E., C. Pacchetti, et al. (1995). "Terguride in stable Parkinson's disease." Funct Neurol **10**(3): 143-146.

Mateo, Z. and J. T. Porter (2007). "Group II metabotropic glutamate receptors inhibit glutamate release at thalamocortical synapses in the developing somatosensory cortex." Neuroscience **146**(3): 1062-1072.

McEvoy, J. P., L. Citrome, et al. (2013). "Effectiveness of lurasidone in patients with schizophrenia or schizoaffective disorder switched from other antipsychotics: a randomized, 6-week, open-label study." J Clin Psychiatry **74**(2): 170-179.

Min, S. K., C. S. Rhee, et al. (1993). "Risperidone versus haloperidol in the treatment of chronic schizophrenic patients: a parallel group double-blind comparative trial." Yonsei Med J **34**(2): 179-190.

Minabe, Y., Y. Shirayama, et al. (2004). "Effect of the acute and chronic administration of the selective 5-HT6 receptor antagonist SB-271046 on the activity of midbrain dopamine neurons in rats: An in vivo electrophysiological study." Synapse **52**(1): 20-28.

Mizuno, Y., K. Hasegawa, et al. (2010). "Clinical efficacy of istradefylline (KW-6002) in Parkinson's disease: a randomized, controlled study." Mov Disord **25**(10): 1437-1443.

Mizuno, Y. and T. Kondo (2013). "Adenosine A2A receptor antagonist istradefylline reduces daily OFF time in Parkinson's disease." Mov Disord **28**(8): 1138-1141.

Mizuno, Y., M. Nomoto, et al. (2014). "Rotigotine vs ropinirole in advanced stage Parkinson's disease: a double-blind study." Parkinsonism Relat Disord **20**(12): 1388-1393.

Moreau, C., A. Delval, et al. (2012). "Methylphenidate for gait hypokinesia and freezing in patients with Parkinson's disease undergoing subthalamic stimulation: a multicentre, parallel, randomised, placebo-controlled trial." Lancet Neurol **11**(7): 589-596.

Moreau, C., A. Delval, et al. (2013). "Memantine for axial signs in Parkinson's disease: a randomised, double-blind, placebo-controlled pilot study." J Neurol Neurosurg Psychiatry **84**(5): 552-555.

Morgante, L., A. Epifanio, et al. (2004). "Quetiapine and clozapine in parkinsonian patients with dopaminergic psychosis." Clin Neuropharmacol **27**(4): 153-156.

Mortimer, A., S. Martin, et al. (2004). "A double-blind, randomized comparative trial of amisulpride versus olanzapine for 6 months in the treatment of schizophrenia." Int Clin Psychopharmacol **19**(2): 63-69.

Mullen, J., M. D. Jibson, et al. (2001). "A comparison of the relative safety, efficacy, and tolerability of quetiapine and risperidone in outpatients with schizophrenia and other psychotic disorders: the quetiapine experience with safety and tolerability (QUEST) study." Clin Ther **23**(11): 1839-1854.

Murata, M., K. Hasegawa, et al. (2007). "Zonisamide improves motor function in Parkinson disease: a randomized, double-blind study." Neurology **68**(1): 45-50.

Na, K. S., C. E. Kim, et al. (2013). "Effectiveness of paliperidone extended-release for patients with schizophrenia: focus on subjective improvement." Hum Psychopharmacol **28**(2): 107-116.

Naber, D., M. Riedel, et al. (2005). "Randomized double blind comparison of olanzapine vs. clozapine on subjective well-being and clinical outcome in patients with schizophrenia." Acta Psychiatr Scand **111**(2): 106-115.

Nakamura, M., M. Ogasa, et al. (2009). "Lurasidone in the treatment of acute schizophrenia: a double-blind, placebo-controlled trial." J Clin Psychiatry **70**(6): 829-836.

Pahwa, R., W. C. Koller, et al. (2007). "Subcutaneous apomorphine in patients with advanced Parkinson's disease: a dose-escalation study with randomized, double-blind, placebo-controlled crossover evaluation of a single dose." J Neurol Sci **258**(1-2): 137-143.

Parikh, V., J. Ji, et al. (2010). "Prefrontal beta2 subunit-containing and alpha7 nicotinic acetylcholine receptors differentially control glutamatergic and cholinergic signaling." J Neurosci **30**(9): 3518-3530.

Parnas, H. (2005). "Depolarization Initiates Phasic Acetylcholine Release by Relief of a Tonic Block Imposed by Presynaptic M2 Muscarinic Receptors." Journal of Neurophysiology **93**(6): 3257-3269.

Perez-Rosello, T., A. Figueroa, et al. (2005). "Cholinergic control of firing pattern and neurotransmission in rat neostriatal projection neurons: role of CaV2.1 and CaV2.2 Ca2+ channels." J Neurophysiol **93**(5): 2507-2519.

Potkin, S. G., R. E. Litman, et al. (2008). "Efficacy of iloperidone in the treatment of schizophrenia: initial phase 3 studies." J Clin Psychopharmacol **28**(2 Suppl 1): S4-11.

Potkin, S. G., A. R. Saha, et al. (2003). "Aripiprazole, an antipsychotic with a novel mechanism of action, and risperidone vs placebo in patients with schizophrenia and schizoaffective disorder." Arch Gen Psychiatry **60**(7): 681-690.

Potkin, S. G., P. J. Weiden, et al. (2009). "Remission in schizophrenia: 196-week, double-blind treatment with ziprasidone vs. haloperidol." Int J Neuropsychopharmacol **12**(9): 1233-1248.

Pourcher, E., H. H. Fernandez, et al. (2012). "Istradefylline for Parkinson's disease patients experiencing motor fluctuations: results of the KW-6002-US-018 study." Parkinsonism Relat Disord **18**(2): 178-184.

Puig, M. V., N. Santana, et al. (2004). "In vivo excitation of GABA interneurons in the medial prefrontal cortex through 5-HT3 receptors." Cereb Cortex **14**(12): 1365-1375.

Putzki, N., M. Maschke, et al. (2002). "Effect of functional NMDA-antagonist flupirtine on automatic postural responses in Parkinson's disease." J Neurol **249**(7): 824-828.

Rascol, O., P. Barone, et al. (2012). "Perampanel in Parkinson disease fluctuations: a double-blind randomized trial with placebo and entacapone." Clin Neuropharmacol **35**(1): 15-20.

Rascol, O., O. Blin, et al. (1999). "ABT-431, a D1 receptor agonist prodrug, has efficacy in Parkinson's disease." Ann Neurol **45**(6): 736-741.

Rascol, O., J. G. Nutt, et al. (2001). "Induction by dopamine D1 receptor agonist ABT-431 of dyskinesia similar to levodopa in patients with Parkinson disease." Arch Neurol **58**(2): 249-254.

Riemer, C., E. Borroni, et al. (2003). "Influence of the 5-HT6 receptor on acetylcholine release in the cortex: pharmacological characterization of 4-(2-bromo-6-pyrrolidin-1-ylpyridine-4-sulfonyl)phenylamine, a potent and selective 5-HT6 receptor antagonist." J Med Chem **46**(7): 1273-1276.

Rosenheck, R., J. Cramer, et al. (1999). "Cost-effectiveness of clozapine in patients with high and low levels of hospital use. Department of Veterans Affairs Cooperative Study Group on Clozapine in Refractory Schizophrenia." Arch Gen Psychiatry **56**(6): 565-572.

Rosenheck, R., L. Dunn, et al. (1999). "Impact of clozapine on negative symptoms and on the deficit syndrome in refractory schizophrenia. Department of Veterans Affairs Cooperative Study Group on Clozapine in Refractory Schizophrenia." Am J Psychiatry **156**(1): 88-93.
Salzman, C., J. Rosenberg, et al. (2007). "Schizophrenia symptoms remain stable during decreases from 2 antipsychotics to aripiprazole." J Clin Psychiatry **68**(6): 970.
Sciarretta, A. and G. Piazzi (2012). "Long-term safety of ziprasidone in schizophrenic patients: an open trial." Eur Rev Med Pharmacol Sci **16**(15): 2113-2119.
Sechter, D., J. Peuskens, et al. (2002). "Amisulpride vs. risperidone in chronic schizophrenia: results of a 6-month double-blind study." Neuropsychopharmacology **27**(6): 1071-1081.
Shen, W., S. E. Hamilton, et al. (2005). "Cholinergic suppression of KCNQ channel currents enhances excitability of striatal medium spiny neurons." J Neurosci **25**(32): 7449-7458.
Simpson, G. M., R. C. Josiassen, et al. (1999). "Double-blind study of clozapine dose response in chronic schizophrenia." Am J Psychiatry **156**(11): 1744-1750.
Spivak, B., E. Shabash, et al. (2003). "The effects of clozapine versus haloperidol on measures of impulsive aggression and suicidality in chronic schizophrenia patients: an open, nonrandomized, 6-month study." J Clin Psychiatry **64**(7): 755-760.
Stacy, M., D. Silver, et al. (2008). "A 12-week, placebo-controlled study (6002-US-006) of istradefylline in Parkinson disease." Neurology **70**(23): 2233-2240.
Stahl, S. M., J. Cucchiaro, et al. (2013). "Effectiveness of lurasidone for patients with schizophrenia following 6 weeks of acute treatment with lurasidone, olanzapine, or placebo: a 6-month, open-label, extension study." J Clin Psychiatry **74**(5): 507-515.
Stocchi, F., R. Borgohain, et al. (2012). "A randomized, double-blind, placebo-controlled trial of safinamide as add-on therapy in early Parkinson's disease patients." Mov Disord **27**(1): 106-112.
Stocchi, F., A. Hsu, et al. (2014). "Comparison of IPX066 with carbidopa-levodopa plus entacapone in advanced PD patients." Parkinsonism Relat Disord **20**(12): 1335-1340.
Storch, A., C. Trenkwalder, et al. (2005). "High-dose treatment with pergolide in Parkinson's disease patients with motor fluctuations and dyskinesias." Parkinsonism Relat Disord **11**(6): 393-398.
Sumiyoshi, T., K. Jayathilake, et al. (2003). "A comparison of two doses of melperone, an atypical antipsychotic drug, in the treatment of schizophrenia." Schizophr Res **62**(1-2): 65-72.
Sumiyoshi, T., K. Jayathilake, et al. (2003). "The effect of melperone, an atypical antipsychotic drug, on cognitive function in schizophrenia." Schizophr Res **59**(1): 7-16.
Suwantamee, J., S. Nidhinandana, et al. (2004). "Efficacy and safety of piribedil in early combination with L-dopa in the treatment of Parkinson's disease: a 6-month open study." J Med Assoc Thai **87**(11): 1293-1300.
Szegedi, A., P. Verweij, et al. (2012). "Meta-analyses of the efficacy of asenapine for acute schizophrenia: comparisons with placebo and other antipsychotics." J Clin Psychiatry **73**(12): 1533-1540.
Tandon, R., R. N. Marcus, et al. (2006). "A prospective, multicenter, randomized, parallel-group, open-label study of aripiprazole in the management of patients with schizophrenia or schizoaffective disorder in general psychiatric practice: Broad Effectiveness Trial With Aripiprazole (BETA)." Schizophr Res **84**(1): 77-89.
Tauscher, J., B. Kufferle, et al. (2002). "Striatal dopamine-2 receptor occupancy as measured with [123I]iodobenzamide and SPECT predicted the occurrence of EPS in patients treated with atypical antipsychotics and haloperidol." Psychopharmacology (Berl) **162**(1): 42-49.
Tollefson, G. D., C. M. Beasley, Jr., et al. (1997). "Olanzapine versus haloperidol in the treatment of schizophrenia and schizoaffective and schizophreniform disorders: results of an international collaborative trial." Am J Psychiatry **154**(4): 457-465.
Tran, P. V., G. D. Tollefson, et al. (1999). "Olanzapine versus haloperidol in the treatment of schizoaffective disorder. Acute and long-term therapy." Br J Psychiatry **174**: 15-22.

Truffinet, P., C. A. Tamminga, et al. (1999). "Placebo-controlled study of the D4/5-HT2A antagonist fananserin in the treatment of schizophrenia." Am J Psychiatry **156**(3): 419-425.
Tseng, K. Y. and P. O'Donnell (2007). "D2 dopamine receptors recruit a GABA component for their attenuation of excitatory synaptic transmission in the adult rat prefrontal cortex." Synapse **61**(10): 843-850.
Van Blercom, N., A. Lasa, et al. (2004). "Effects of gabapentin on the motor response to levodopa: a double-blind, placebo-controlled, crossover study in patients with complicated Parkinson disease." Clin Neuropharmacol **27**(3): 124-128.
van Kammen, D. P., J. P. McEvoy, et al. (1996). "A randomized, controlled, dose-ranging trial of sertindole in patients with schizophrenia." Psychopharmacology (Berl) **124**(1-2): 168-175.
Vartiainen, H., E. Leinonen, et al. (1993). "A long-term study of remoxipride in chronic schizophrenic patients." Acta Psychiatr Scand **87**(2): 114-117.
Volavka, J., P. Czobor, et al. (2002). "Clozapine, olanzapine, risperidone, and haloperidol in the treatment of patients with chronic schizophrenia and schizoaffective disorder." Am J Psychiatry **159**(2): 255-262.
Walinder, J. and A. C. Holm (1990). "Experiences of long-term treatment with remoxipride: efficacy and tolerability." Acta Psychiatr Scand Suppl **358**: 158-163.
Wang, Y., S. Sun, et al. (2014). "The efficacy and safety of pramipexole ER versus IR in Chinese patients with Parkinson's disease: a randomized, double-blind, double-dummy, parallel-group study." Transl Neurodegener **3**: 11.
Werneck, A. L., A. L. Rosso, et al. (2009). "The use of an antagonist 5-HT2a/c for depression and motor function in Parkinson' disease." Arq Neuropsiquiatr **67**(2B): 407-412.
Wetzel, H., G. Grunder, et al. (1998). "Amisulpride versus flupentixol in schizophrenia with predominantly positive symptomatology -- a double-blind controlled study comparing a selective D2-like antagonist to a mixed D1-/D2-like antagonist. The Amisulpride Study Group." Psychopharmacology (Berl) **137**(3): 223-232.
Wills, A. M., S. Eberly, et al. (2013). "Caffeine consumption and risk of dyskinesia in CALM-PD." Mov Disord **28**(3): 380-383.
Yuen, E. Y. and Z. Yan (2009). "Dopamine D4 receptors regulate AMPA receptor trafficking and glutamatergic transmission in GABAergic interneurons of prefrontal cortex." J Neurosci **29**(2): 550-562.
Zhang, W., M. Yamada, et al. (2002). "Multiple muscarinic acetylcholine receptor subtypes modulate striatal dopamine release, as studied with M1-M5 muscarinic receptor knock-out mice." J Neurosci **22**(15): 6347-6352.
Ziegler, M., A. Castro-Caldas, et al. (2003). "Efficacy of piribedil as early combination to levodopa in patients with stable Parkinson's disease: a 6-month, randomized, placebo-controlled study." Mov Disord **18**(4): 418-425.
Zimbroff, D. L., J. M. Kane, et al. (1997). "Controlled, dose-response study of sertindole and haloperidol in the treatment of schizophrenia. Sertindole Study Group." Am J Psychiatry **154**(6): 782-791.
Zipursky, R. B., B. K. Christensen, et al. (2005). "Treatment response to olanzapine and haloperidol and its association with dopamine D receptor occupancy in first-episode psychosis." Can J Psychiatry **50**(8): 462-469.
